# Functional diversity of secreted cestode Kunitz proteins: Inhibition of serine peptidases and blockade of cation channels

**DOI:** 10.1371/journal.ppat.1006169

**Published:** 2017-02-13

**Authors:** Martín Fló, Mariana Margenat, Leonardo Pellizza, Martín Graña, Rosario Durán, Adriana Báez, Emilio Salceda, Enrique Soto, Beatriz Alvarez, Cecilia Fernández

**Affiliations:** 1 Cátedra de Inmunología, Facultad de Química, Universidad de la República, Montevideo, Uruguay; 2 Laboratorio de Enzimología, Facultad de Ciencias, Universidad de la República, Montevideo, Uruguay; 3 Unidad de Bioinformática, Institut Pasteur de Montevideo, Montevideo, Uruguay; 4 Unidad de Bioquímica y Proteómica Analíticas, Institut Pasteur de Montevideo and Instituto de Investigaciones Biológicas Clemente Estable, Montevideo, Uruguay; 5 Instituto de Fisiología, Benemérita Universidad Autónoma de Puebla, Puebla, México; George Washington University School of Medicine and Health Sciences, UNITED STATES

## Abstract

We previously reported a multigene family of monodomain Kunitz proteins from *Echinococcus granulosus* (*Eg*KU-1-*Eg*KU-8), and provided evidence that some *Eg*KUs are secreted by larval worms to the host interface. In addition, functional studies and homology modeling suggested that, similar to monodomain Kunitz families present in animal venoms, the *E*. *granulosus* family could include peptidase inhibitors as well as channel blockers. Using enzyme kinetics and whole-cell patch-clamp, we now demonstrate that the *Eg*KUs are indeed functionally diverse. In fact, most of them behaved as high affinity inhibitors of either chymotrypsin (*Eg*KU-2-*Eg*KU-3) or trypsin (*Eg*KU-5-*Eg*KU-8). In contrast, the close paralogs *Eg*KU-1 and *Eg*KU-4 blocked voltage-dependent potassium channels (K_v_); and also pH-dependent sodium channels (ASICs), while showing null (*Eg*KU-1) or marginal (*Eg*KU-4) peptidase inhibitory activity. We also confirmed the presence of *Eg*KUs in secretions from other parasite stages, notably from adult worms and metacestodes. Interestingly, data from genome projects reveal that at least eight additional monodomain Kunitz proteins are encoded in the genome; that particular *Eg*KUs are up-regulated in various stages; and that analogous Kunitz families exist in other medically important cestodes, but not in trematodes. Members of this expanded family of secreted cestode proteins thus have the potential to block, through high affinity interactions, the function of host counterparts (either peptidases or cation channels) and contribute to the establishment and persistence of infection. From a more general perspective, our results confirm that multigene families of Kunitz inhibitors from parasite secretions and animal venoms display a similar functional diversity and thus, that host-parasite co-evolution may also drive the emergence of a new function associated with the Kunitz scaffold.

## Author summary

Parasite secretions are key players at host-parasite interfaces: parasite establishment and persistence rely, to a great extent, on interactions between these molecules and their host counterparts. We present the functional characterization of a multigene family of secreted Kunitz proteins from the cestode *Echinococcus granulosus*. Kunitz proteins are a class of metazoan high affinity serine peptidase inhibitors. In addition, families of Kunitz proteins are frequent components of animal venoms; besides inhibiting peptidases, some of these “Kunitz toxins” block cation channels and thus, provide a remarkable example of protein evolution where natural selection has driven the emergence of a new function associated with the same molecular scaffold. Using enzyme kinetics and electrophysiological assays, we demonstrated that the *E*. *granulosus* Kunitz family includes peptidase inhibitors as well as channel blockers. This diversity highlights an interesting similarity between animal toxins and parasite secretions that had not been previously described. Furthermore, the presence of analogous families of Kunitz proteins appears to be a distinctive trait of cestode genomes. We thus propose that these molecules contribute to a successful infection acting at the parasite-host interface. In addition, because they bear a strong specificity towards their targets, they are uniquely suited for the development of pharmaceuticals.

## Introduction

Cestodes are a neglected group of platyhelminth parasites, despite causing chronic infections to humans and domestic animals worldwide [1]. Together with other researchers around the world [2], we have been using *Echinococcus granulosus* as a model to study the molecular basis of the host-parasite cross-talk during cestode infections [3,4,5]. *E*. *granulosus* is the agent of cystic echinococcosis, a medically and economically important worldwide zoonosis, with endemic foci in Central Asia, China, South America and Africa [6]. Like all *taeniid* cestodes, it has a life cycle involving two mammals: a non carnivore intermediate host (harboring the larva) and a carnivore definitive host (harboring the hermaphroditic adult). Intermediate hosts (ungulates such as sheep, cattle and pigs; and, accidentally, also humans) become infected by ingestion of eggs containing oncospheres that develop at visceral sites into bladder-like metacestodes (hydatid cysts). These latter are bounded by a wall whose inner germinal layer gives rise to larval worms (protoscoleces) by asexual budding; protoscoleces are bathed in hydatid fluid that includes host plasmatic proteins and parasite secretions. Infection in the definitive host (always a canid, most often dogs) arises from ingestion of protoscoleces that, upon activation by contact with stomach acid, enzymes and bile acids, evaginate and attach to the mucosa of the duodenum, where they develop into adult tapeworms that can reside in the gut for long periods without causing any apparent damage [7]. Specific anatomical structures allow such a close contact at the canid-worm interface that *E*. *granulosus* has been regarded as both a tissue and a luminal parasite [8]. The molecular mechanisms underlying its successful establishment and persistence in the hostile environment of the dog duodenum are unknown.

With the aim of identifying molecules participating in the *E*. *granulosus*–dog cross-talk, we surveyed the genes expressed by protoscoleces and pepsin/H^+^-treated protoscoleces. We thus identified a multigene family of Kunitz-type inhibitors (*Eg*KUs). These molecules were associated mostly with treated protoscoleces, suggesting that they play roles at the initial phases of infection [3]. Kunitz inhibitors are a class of metazoan serine peptidase inhibitors, whose prototype is the bovine pancreatic inhibitor of trypsin (BPTI; family I2 of the MEROPS database; http://merops.sanger.ac.uk/) [9]. They are competitive inhibitors acting in a substrate-like manner, that form very stable complexes of 1:1 stoichiometry with their target enzymes, devoid of activity. The interaction between the enzyme and the inhibitor is highly dependent on the residue located at the position P1 of the antipeptidase loop (position 15 of mature BPTI) [10]. In addition, families of Kunitz inhibitors are frequent components of the saliva and secretions from hematophagous animals and also of animal venoms. These “Kunitz-type toxins” have been described in the venoms from snakes [11], sea anemones [12,13], cone snails [14], spiders [15], scorpions [16,17] as well as in the saliva of blood-sucking arthropods [18,19] and in the secretions of hematophagous nematodes [20]. Interestingly, besides inhibiting peptidases, some Kunitz toxins, until now described only in animal venoms, block various types of cation channels. Furthermore, some act solely as channel blockers. A set of neurotoxins present in the venoms of mamba snakes (‘‘dendrotoxins”), whose function is to paralyze the prey, is the best known example [21].

We previously reported the molecular features of eight *Eg*KUs (that we named *Eg*KU-1-*Eg*KU-8) and provided evidence that some of them (notably, *Eg*KU-3 and *Eg*KU-8) are secreted by protoscoleces. Although diverse, these *Eg*KUs were found to group into three pairs of close paralogs (*Eg*KU-1/*Eg*KU-4; *Eg*KU-3/*Eg*KU-8; *Eg*KU-6/*Eg*KU-7), which would be the products of recent gene duplications. In addition, we carried out detailed kinetic studies with native *Eg*KU-1 and *Eg*KU-8 purified from protoscoleces that revealed their possible functionalities. *Eg*KU-8 behaved as a slow, tight-binding inhibitor of trypsins, with global inhibition constants (*K*_I_^*^) in the 10^−11^ M range, and interacted with enzymes through a mechanism involving two reversible steps; an initial relatively fast formation of an enzyme-inhibitor complex followed by a slow transition to a tight complex. In sharp contrast, *Eg*KU-1 did not inhibit any of the assayed peptidases. Interestingly, molecular modeling revealed that structural elements associated with activity in Kunitz cation-channel blockers are also present in *Eg*KU-1. Indeed, α-dendrotoxin (α-DTX), a well characterized blocker of specific voltage-activated K^+^-channels (K_v_) [21], was—at the time—the best overall template of *Eg*KU-1; and several amino acids important for toxin activity were found to be conserved in the consensus model of the parasite molecule, supporting the notion that it is a putative cation channel blocker. Presumed orthologs of the *Eg*KUs (peptidase inhibitors as well as channel blockers) were also found to be present in the transcriptomes from the other medically important cestodes (notably, *E*. *multilocularis* and *Taenia solium*, the agents of alveolar echinococcosis and cysticercosis, respectively), indicating that families of monodomain Kunitz inhibitors are also present in closely related organisms [3].

In this article, we characterize the activity of *Eg*KU-1–*Eg*KU-8 using enzyme kinetics and whole-cell patch clamp assays. We thus demonstrate that the *E*. *granulosus* Kunitz family is indeed functionally diverse. On the one hand, we show that all but *Eg*KU-1 and *Eg*KU-4 behave as high affinity inhibitors of either chymotrypsin or trypsin. On the other hand, patch-clamp assays on rat dorsal root ganglion (DRG) neurons confirmed that *Eg*KU-1, and also its close paralog *Eg*KU-4, block K_v_. Furthermore, the two proteins also block pH-dependent sodium channels (acid sensing ion channels, ASICs), a previously unreported activity for Kunitz inhibitors, that we recently described for α-DTX [22]. In addition, we provide further evidence of the presence of *Eg*KUs in parasite secretions. We discuss the significance of these results taking into account available genomic and transcriptomic data from *E*. *granulosus* and related cestodes.

## Results

### Except for *Eg*KU-1 and *Eg*KU-4, the *Eg*KUs are high affinity inhibitors of serine peptidases

**Fig 1:**
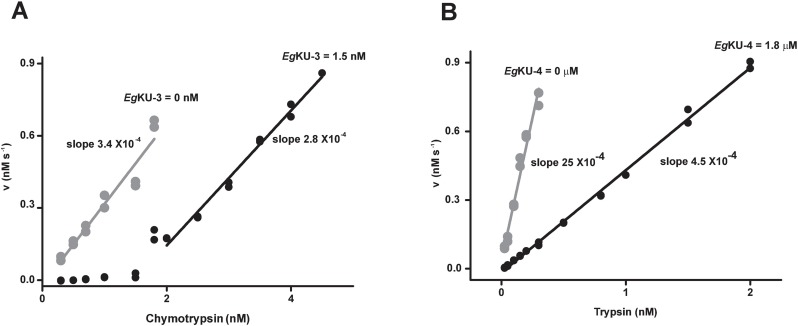
Titration assays of recombinant *Eg*KUs: results for *Eg*KU-3 and *Eg*KU-4. Increasing concentrations of bovine chymotrypsin or trypsin were pre-incubated with fixed amounts of recombinant *Eg*KU-3 (A) or *Eg*KU-4 (B), respectively, and mixed with the corresponding enzyme substrate. The plots show the initial steady-state rate of substrate hydrolysis for each enzyme concentration; the activity in the absence of inhibitor is indicated in grey. (A) *Eg*KU-3 is a high affinity inhibitor of chymotrypsin. Note that the slope at the enzyme concentrations for which activity is detected compares very well with the slope in the absence of inhibitor. The x-intercept of this plot (1.5 nM) represents the enzyme concentration interacting with 1.5 nM of *Eg*KU-3. Thus, *Eg*KU-3 inhibits chymotrypsin with a 1:1 stoichiometry. (B) *Eg*KU-4 is a low affinity inhibitor of trypsin. Note that trypsin activity is detected all over the assayed enzyme range in the presence of an inhibitor concentration 1000-fold higher than the peptidase concentration. Representative results are shown. Experiments with *Eg*KU-3 and *Eg*KU-4 were carried out five and two independent times, respectively. Within each experiment, measurements were performed in duplicates.

In our previous study, working with native *Eg*KU-1 and *Eg*KU-8, we demonstrated that *Eg*KU-8 is a high affinity inhibitor of trypsins, whereas *Eg*KU-1 did not inhibit any of the assayed peptidases [3]. To further advance in the functional characterization of the family, we prepared recombinants of the eight *Eg*KUs. We carried out a preliminary screening of the serine peptidase inhibition activity of recombinant *Eg*KU-2–*Eg*KU-7. Of note, in the case of *Eg*KU-8 both the native inhibitor and the recombinant protein behaved similarly (*K*_I_^*^ 60 ± 13 *versus* 50 ± 10 pM, for native and recombinant *Eg*KU-8, respectively). Using pancreatic enzymes, we analyzed whether the *Eg*KUs showed the inhibition profiles that may be predicted from the respective amino acid at position P1: *Eg*KU-2 (Trp in P1) and *Eg*KU-3 (Leu in P1) are candidate chymotrypsin inhibitors, whereas *Eg*KU-4-*Eg*KU-7 (Arg in P1) are predicted to inhibit trypsin. All six *Eg*KUs showed the expected activities. We subsequently performed titration assays to analyze whether they behaved as high affinity inhibitors. These studies indicated that, except for *Eg*KU-4, the close paralog of *Eg*KU-1, the *Eg*KUs are high affinity inhibitors of bovine chymotrypsin (*Eg*KU-2 and *Eg*KU-3) or trypsin (*Eg*KU-5-*Eg*KU-8) (Table 1 and Fig 1). In view of these results, we further characterized the inhibitory activity of *Eg*KU-3, the closest paralog of *Eg*KU-8.

**Table 1:** Screening of serine peptidase inhibitory activity of *Eg*KUs.

	P1^a^	Trypsin^b^	Chymotrypsin^b^
***Eg*KU-1**^c^	Q	NI^d^	NI^d^
***Eg*KU-2**	W	ND^e^	**High**
***Eg*KU-3**	L	NI^d^	**High**
***Eg*KU-4**	R	Low	ND^e^
***Eg*KU-5**	R	**High**	ND^e^
***Eg*KU-6**	R	**High**	ND^e^
***Eg*KU-7**	R	**High**	ND^e^
***Eg*KU-8**^c^	R	**High**	Low

The activity was considered “high” when curvatures were observed in titration assays (initial steady-state rate *versus* enzyme concentration plots) and the *K*_I_^*^ values were < 10^−10^ M. The activity was considered “low” when titration plots were linear and the *K*_I_^*^ values were > 10^−9^ M. Refer to Fig 1 and the text for further details.

^a^Amino acid at position 15 (numbering as per mature BPTI), corresponding to the active site of serine peptidase inhibitors.

^b^The assays were carried out with cationic trypsin and chymotrypsin A from bovine pancreas.

^c^Data with recombinant *Eg*KU-1 and *Eg*KU-8 reproduced those obtained with the native proteins ([3]; see the text for further details).

^d^NI, not inhibited.

^e^ND, not determined.

### *Eg*KU-3 inhibits chymotrypsin with high affinity through a two-step mechanism

**Fig 2:**
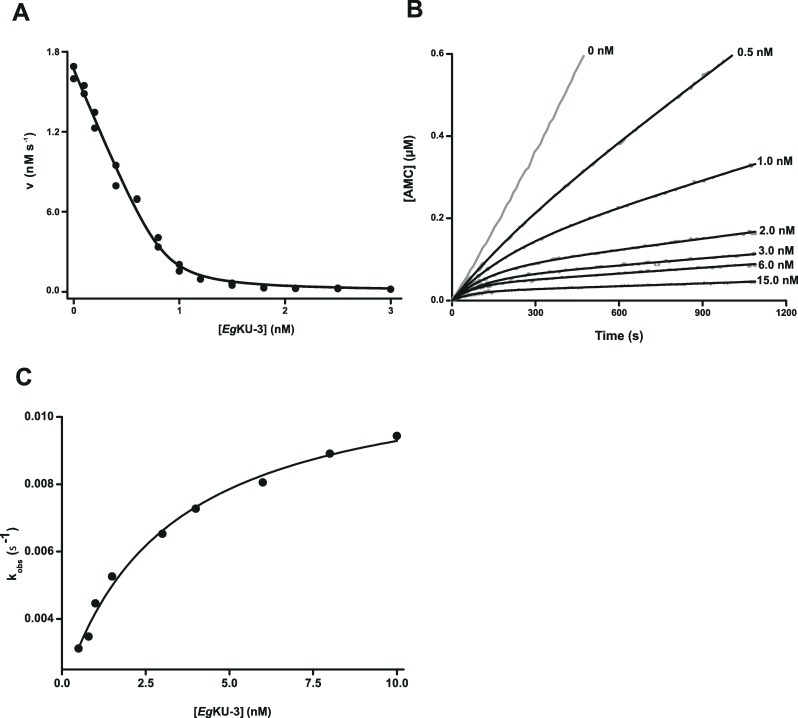
Inhibition studies with *Eg*KU-3: results for bovine chymotrypsin A. (A) Enzyme inhibition. The enzyme (1 nM) was preincubated for 15 min with *Eg*KU-3 (0.1–3.0 nM) and mixed with substrate (Suc-Ala-Ala-Pro-Phe-AMC, 5 μM) in 50 mM Tris-HCl, pH 8.0, 0.01% Triton X-100, at 37°C. Initial steady-state rate measurements were performed in duplicates and the experiment was repeated 5 independent times. A representative experiment is shown. *K*_I_^*^_app_ values at equilibrium were determined using Eq (1) for tight binding inhibitors as described in Materials and Methods. The solid line represents the best fit to this equation. (B) Representative progress curves for the inhibition. The enzyme (1 nM) was added to reaction mixtures containing the substrate (Suc-Ala-Ala-Pro-Phe-AMC, 5 μM) and increasing concentrations of *Eg*KU-3 (0, 0.5, 1, 2, 3, 6, and 15 nM, gray traces) in 50 mM Tris-HCl, pH 8.0, 0.01% Triton X-100, at 37°C. The black traces represent the best fit to Eq 3, from which *k*_obs_ were obtained. (C) Dependence of *k*_obs_ on the concentration of inhibitor. The enzyme was added to reaction mixtures containing the substrate (Suc-Ala-Ala-Pro-Phe-AMC, 5 μM) and increasing concentration of *Eg*KU-3 in 50 mM Tris-HCl, pH 8.0, 0.01% Triton X-100, at 37°C. The enzyme concentrations were: 1 nM for 0.5–3 nM of *Eg*KU-3, 2 nM for 3–6 nM of *Eg*KU-3, and 3 nM for 6–10 nM of *Eg*KU-3. *k*_obs_ values were obtained from time course experiments according to Eq 3 and correspond to the average of at least two time courses. The black trace represents the best fit to Eq 5 in agreement with Eq 4. The experiment was repeated 3 independent times.

We carried out kinetic studies using bovine chymotrypsin A and also chymotrypsin purified from dog pancreas, *i*. *e*. chymotrypsin B (chymotrypsin A is absent from dogs, see S01.001 at MEROPS—http://merops.sanger.ac.uk). *Eg*KU-3 inhibited with high affinity both peptidases; Fig 2A shows a representative experiment with the bovine enzyme and Table 2 the global inhibition constants calculated for the two chymotrypsins. The values of *K*_I_^*^ were of the same order (53 ± 19 and 84 ± 49 pM for the bovine and canine enzymes, respectively) indicating no bias in specificity towards any of them. *Eg*KU-3 also inhibited elastase, although with substantially lower affinity than chymotrypsins (*K*_I_^*^ of 5 ± 2 nM).

**Table 2:** Global inhibition constants (*K*_I_^*^) of *Eg*KU-3, *Eg*KU-4 and *Eg*KU-8 acting on pancreatic serine peptidases.

*K*_I_^*^(pM)^a^				
	Trypsin^b^	Chymotrypsin A^b^	Chymotrypsin B^b^	Elastase^b^
***Eg*KU-3**	NI^c^	53 ± 19	84 ± 49	(5 ± 2) x 10^3^
***Eg*KU-4**	(47 ± 2) x 10^3^	ND^d^	ND^d^	ND^d^
***Eg*KU-8**^e^	60 ± 13	(2.0 ± 0.2) x 10^3^	NI^c^	NI^c^
**BPTI**^f^	0.6	1.3 x 10^3^	NI^g^	2.6 x 10^6^

^a^*K*_I_^*^, the global equilibrium dissociation constants, were calculated from inhibition assays (see Fig 2A) according to Eq 1 for tight-binding inhibitors and minimally corrected for the effect of substrate concentration according to Eq 2. Values correspond to averages of independent experiments ± the standard error (n ≥ 3, except for *Eg*KU-4 in which case n = 2).

^b^Bovine cationic trypsin and chymotrypsin A, canine chymotrypsin B, and porcine elastase were used as target enzymes.

^c^NI, not inhibited.

^d^ND, not determined.

^e^Data are from native *Eg*KU-8, as reported in [3].

^f^ Data are from MEROPS (I02.001).

^g^The lack of inhibition refers to bovine chymotrypsin B [66].

In order to study the inhibition mechanism of *Eg*KU-3 towards chymotrypsins, we carried out time course experiments with chymotrypsin A. The progress curves for the inhibition (Fig 2B) indicated that the enzyme-inhibitor complex reaches equilibrium in a time scale of minutes and that *Eg*KU-3 is a slow-binding inhibitor as defined by Morrison [23]. The interaction of *Eg*KU-3 with chymotrypsin was reversible, since progress curves reached appreciable slopes even at higher than stoichiometric inhibitor concentrations. This is the expected behavior for Kunitz-type inhibitors [10] and the one observed for *Eg*KU-8 [3]. Similarly, the plot of the apparent rate constant (*k*_obs_) *versus Eg*KU-3 concentration was hyperbolical (Fig 2C), in accordance with a mechanism involving two steps, a fast initial binding of the inhibitor to the target enzyme followed by a slow transition [24]. The kinetic constants of *Eg*KU-3 binding to chymotrypsin obtained from analyses of the progress curves are shown in Table 3. Note that the value of *K*_I_^*^ calculated from the kinetic constants compared very well with the value obtained through the fit of steady-state rate *versus* inhibitor concentration data to the Morrison equation (Table 2).

**Table 3:** Inhibitory kinetics of *Eg*KU-3 on bovine chymotrypsin A.

Kinetic constant	Chymotrypsin
***k***_**2**_^a^	(1.2 ± 0.2) x 10^−2^ s^-1^
***K***_**I**_^a^	3.2 ± 1.2 nM
***k***_**2**_**/*K***_**I**_^a^	(3.8 ± 2.0) x 10^6^ M^-1^ s^-1^
***k***_**-2**_^b^	(2.8 ± 2.3) x 10^−4^ s^-1^
***K***_I_^*^^c^	73 ± 30 pM

^a^*k*_2_, *K*_I_ and *k*_2_/*K*_I_ were calculated from time course experiments (see Fig 2B and 2C) according to the fit to Eq 5 of *k*_obs_ *versus* [I] plots. Values are averages of independent measurements ± the standard error (n ≥ 2).

^b^*k*_-2_ was calculated from time course experiments according to Eq 6. The value is the average of independent measurements ± the standard deviation (n = 15).

^c^*K*_I_^*^ was calculated from Eq 7 using the values of *k*_2_, *K*_I_ and *k*_-2_ obtained from time course experiments. The value is the average of independent measurements ± the standard error (n ≥ 3).

### *Eg*KU-1 and *Eg*KU-4 block voltage-activated potassium channels (K_v_)

**Fig 3:**
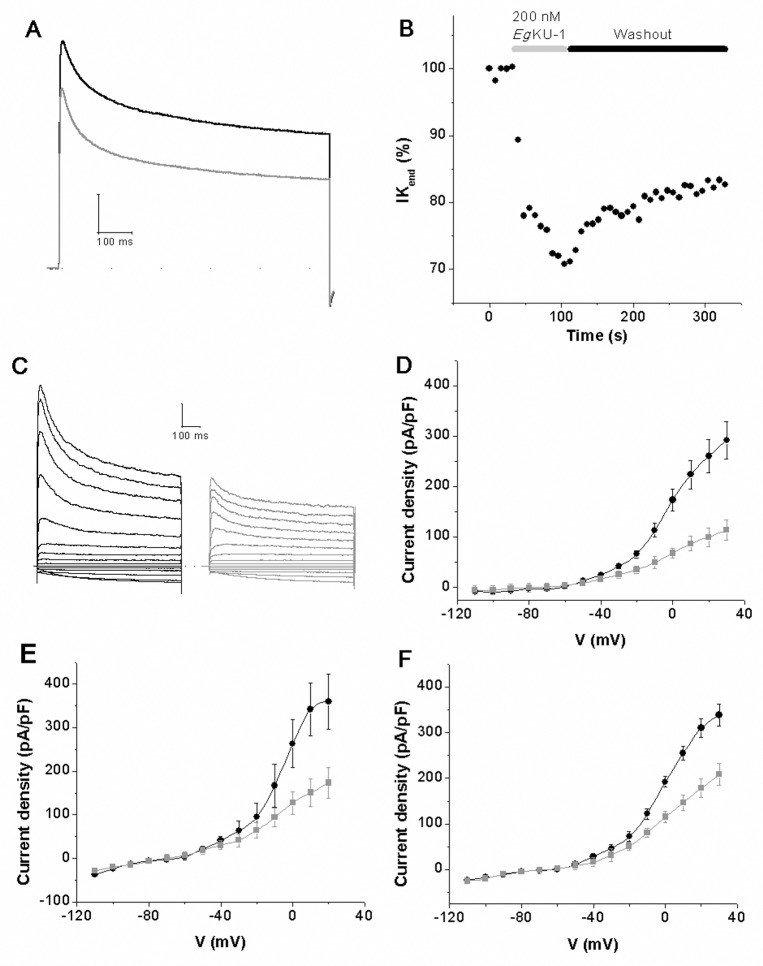
Inhibition studies with *Eg*KU-1 and *Eg*KU-4: results for K_v_ from DRG neurons. Representative experiments showing that recombinant *Eg*KU-1 (200 nM) (A) blocks voltage dependent K^+^ currents elicited by a pulse of -100 to 0 mV during 800 ms (holding potential V_h_ = -60 mV); and (B) that the inhibition effect is only partially reversible after washout of the inhibitor. (C)–(F) Effect of the *Eg*KUs on K^+^ currents activated by increasing voltage pulses. The K^+^ currents were recorded following stepwise increments of 10 mV of the membrane voltage between -110 and 30 mV from a holding potential of -60 mV. Recordings showing the effect of recombinant *Eg*KU-1 (200 nM) are shown in (C) and the current-voltage relationship of these traces in (D). Similar analyses with native *Eg*KU-1 (100 nM) and recombinant *Eg*KU-4 (200 nM) are shown in (E) and (F), respectively. The black traces correspond to control conditions and the gray ones after *Eg*KU perfusion. Note that the effects of native and recombinant *Eg*KU-1 are of the same order.

**Fig 4:**
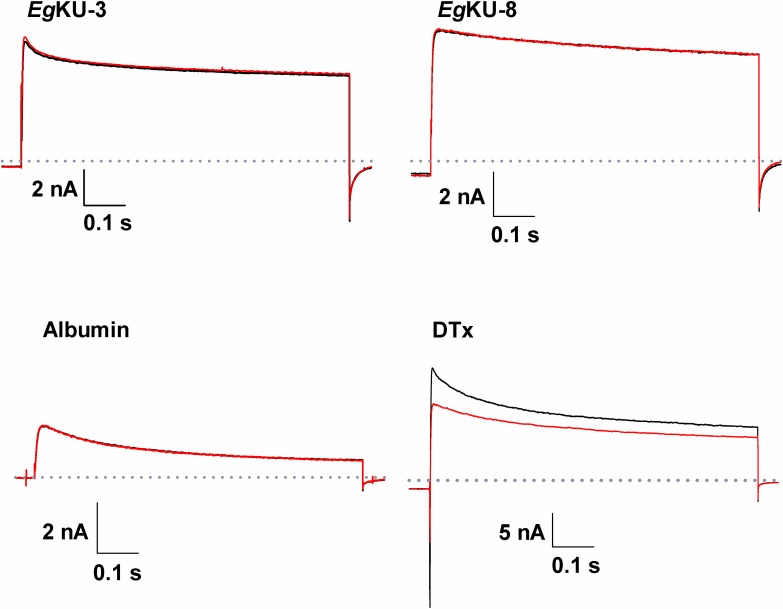
Studies with *Eg*KU-3 and *Eg*KU-8 on total K^+^ currents from DRG neurons. Representative experiments showing that recombinant *Eg*KU-3 and *Eg*KU-8 (1 μM) do not block voltage-dependent K^+^ currents elicited by a pulse of -100 to 0 mV during 800 ms (V_h_ = -60 mV). The superimposed traces correspond to control recordings (black) and records after the perfusion of each *Eg*KU (red). Positive and negative controls were carried out in parallel, using α-DTX (100 nM) and albumin (15 μM), respectively.

As already mentioned, our results indicate that the paralogs *Eg*KU-1/*Eg*KU-4 do not show the typical serine peptidase inhibitory activity of Kunitz-type inhibitors. In fact, *Eg*KU-1 did not inhibit any assayed peptidase [3]; whereas *Eg*KU-4 inhibited trypsin albeit with low affinity, with a *K*_I_^*^ of 47 ± 2 nM, *i*. *e*. 1000-fold higher than the *K*_I_^*^ of *Eg*KU-3 and *Eg*KU-8 *versus* their target enzymes (Table 2). In view of these results and taking into account the structural similarity between *Eg*KU-1 and α-DTX [3], we analyzed whether *Eg*KU-1 and *Eg*KU-4 acted on K_v_ using whole-cell patch-clamp assays on neurons isolated from DRG. Both *Eg*KUs inhibited K_v_; Fig 3A illustrates the effect of recombinant *Eg*KU-1. The blockade was more pronounced over the steady-state component of the current than over the peak current: 25 ± 11% *versus* 20 ± 14% and 27 ± 12% *versus* 23 ± 7% for 200 nM of recombinant *Eg*KU-1 and *Eg*KU-4, respectively (n = 7). We also tested the activity of native *Eg*KU-1 and verified that the recombinant inhibitor reproduced reasonably well the behavior of the native inhibitor (100 nM of native *Eg*KU-1 blocked the steady-state current by 19% and the peak current by 10%; n = 4). Thus, the effect appeared to be stronger on the currents at the end of the pulse (accounting for non inactivating -delayed-rectifier- K^+^ currents, IK_DR_) than on those at the beginning of the pulse (corresponding to fast -transient A-type- K^+^ currents, IK_A_). The effect was only partially reversible because about 60% persisted 3 min after washing (Fig 3B). In addition, it was clearly observed on the currents elicited over -40 mV, as highlighted by the activity profile of the *Eg*KUs over the K^+^ currents activated by different voltages (Fig 3D–3F). In contrast, the perfusion of 1 μM *Eg*KU-8 (n = 7) produced no significant changes in the peak amplitude (2.6 ± 3.2%, P = 0.20) or the steady-state current (1.1 ± 3.5%, P = 0.38), whereas 1 μM of *Eg*KU-3 (n = 10) produced a slight non-significant reduction of the peak current (6.3 ± 3.8%, P = 0.13) and had no effect on the steady-state current (4.1 ± 4.5%, P = 0.13) (Fig 4). We also analyzed the effect of *Eg*KU-1 and *Eg*KU-4 on voltage-activated sodium channels (Na_v_) and observed no effect (S1 Fig).

**Fig 5:**
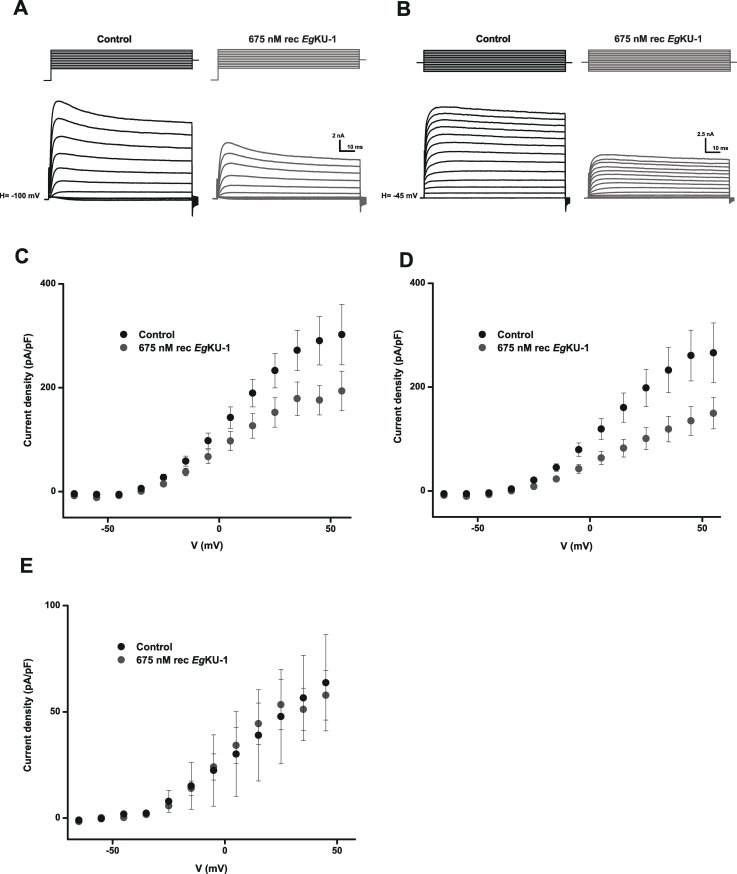
Inhibition studies with *Eg*KU-1 on isolated K^+^ currents from DRG neurons. Blocking effect of recombinant *Eg*KU-1 (675 nM) on isolated K^+^ currents activated by increasing voltage pulses. (A) Voltage-dependent K^+^ currents (fast -transient A-type- currents, IK_A_; as well as non inactivating -delayed-rectifier- currents, IK_DR_) were recorded from a holding potential of -100 mV, following stepwise increments of 10 mV of the membrane voltage, between -65 and 55 mV. (B) IK_DR_ currents were similarly recorded from a holding potential of -45mV, so as to inactivate IK_A_ currents. (C) and (D) are the corresponding current-voltage plots of (A) and (B), whereas (E) is the current-voltage plot accounting for IK_A_ currents and was obtained by subtracting (D) from (C).

**Fig 6:**
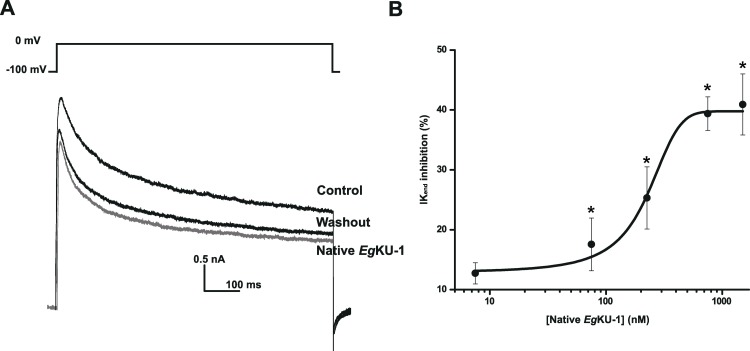
Concentration-response analysis of native *Eg*KU-1 on total K^+^ currents from DRG neurons. (A) Representative traces showing total K^+^ currents elicited by a voltage pulse of -100 to 0 mV during 1000 ms (as indicated above the current trace) under control conditions, after 1 min perfusion of 200 nM of native *Eg*KU-1 and after washing. (B) Concentration-response analysis of *Eg*KU-1 inhibitory effect on K^+^ currents, measured at the end of the voltage pulse, on the steady-state component of the current. The black line shows the best fit to the dose-response equation, from which the IC_50_ was calculated (216 ± 26 nM). The data correspond to the mean ± standard error (n = 5 in all cases). The asterisks indicate Student’s *t*-test significance with respect to the effect in the absence of inhibitor (P ≤ 0.05).

Subsequently, we further characterized the effect of *Eg*KU-1 and *Eg*KU-4 on K_v_. For this purpose, we recorded the currents after a pre-pulse of -120 mV to activate all voltage-dependent K^+^ currents, transient IK_A_ as well as slow-inactivating IK_DR_; and also those remaining when the pre-pulse was of -45 mV, voltage at which IK_A_ is inactivated. Thus, the second recording corresponded to IK_DR_; whereas IK_A_ could be deduced by subtracting IK_DR_ from the first recording. A representative experiment with recombinant *Eg*KU-1 is shown in Fig 5.This setup allowed us to analyze the effect of the inhibitors on total K^+^ currents (Fig 5A and 5C) as well as on both types of isolated K^+^ currents, IK_DR_ (Fig 5B and 5D) and IK_A_ (Fig 5E). As anticipated by the previous experiment, *Eg*KU-1 principally affected IK_DR_, with virtually no effect on IK_A_ (Fig 5D *versus* 5E). Finally, we studied the concentration-response relationship for native *Eg*KU-1 and estimated an IC_50_ of about 200 nM when acting on all K^+^ currents activated by a pulse of -100 to 0 mV (Fig 6). Although we did not determine the IC_50_ for *Eg*KU-4, its behavior was similar to the one of *Eg*KU-1.

### *Eg*KU-1 and *Eg*KU-4 block Acid Sensing Ion Channels (ASICs)

**Fig 7:**
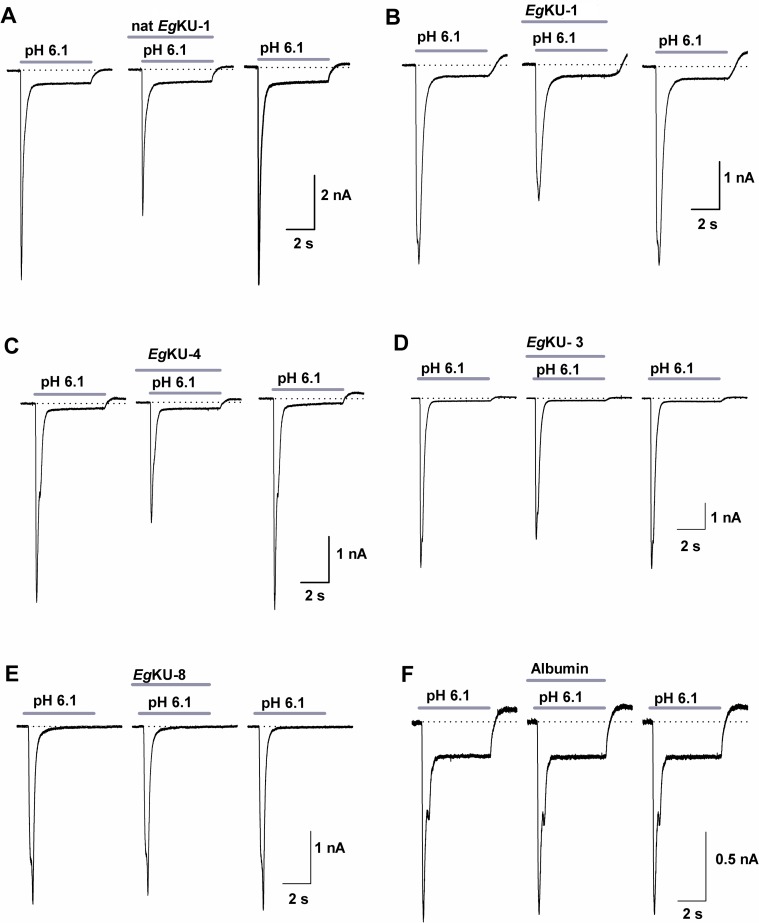
Inhibition studies with *Eg*KU-1 and *Eg*KU-4: results for ASIC currents from DRG neurons. (A-C) Representative traces showing the acid (pH 6.1, 5 s) activated current under control conditions (left), after sustained (25 s) perfusion of 30 nM of each *Eg*KU (center) and after 1 min washout of the inhibitors (right). Note that *Eg*KU-1 and *Eg*KU-4 reduced the amplitude of the Na^+^ current, that recombinant *Eg*KU-1 reproduced the effect of the native inhibitor and that the recovery after washout was higher than 90% in all cases. (D-E) Representative traces from analogous assays with 30 nM of *Eg*KU-3 and *Eg*KU-8. The slight decrement of the current amplitude induced by *Eg*KU-3 was significant (see the text for further details); *Eg*KU-8 had no effect. (F) Albumin (15 μM) was used as negative control. Calibration in each case applies to the control, effect and washout recordings of each panel.

**Fig 8:**
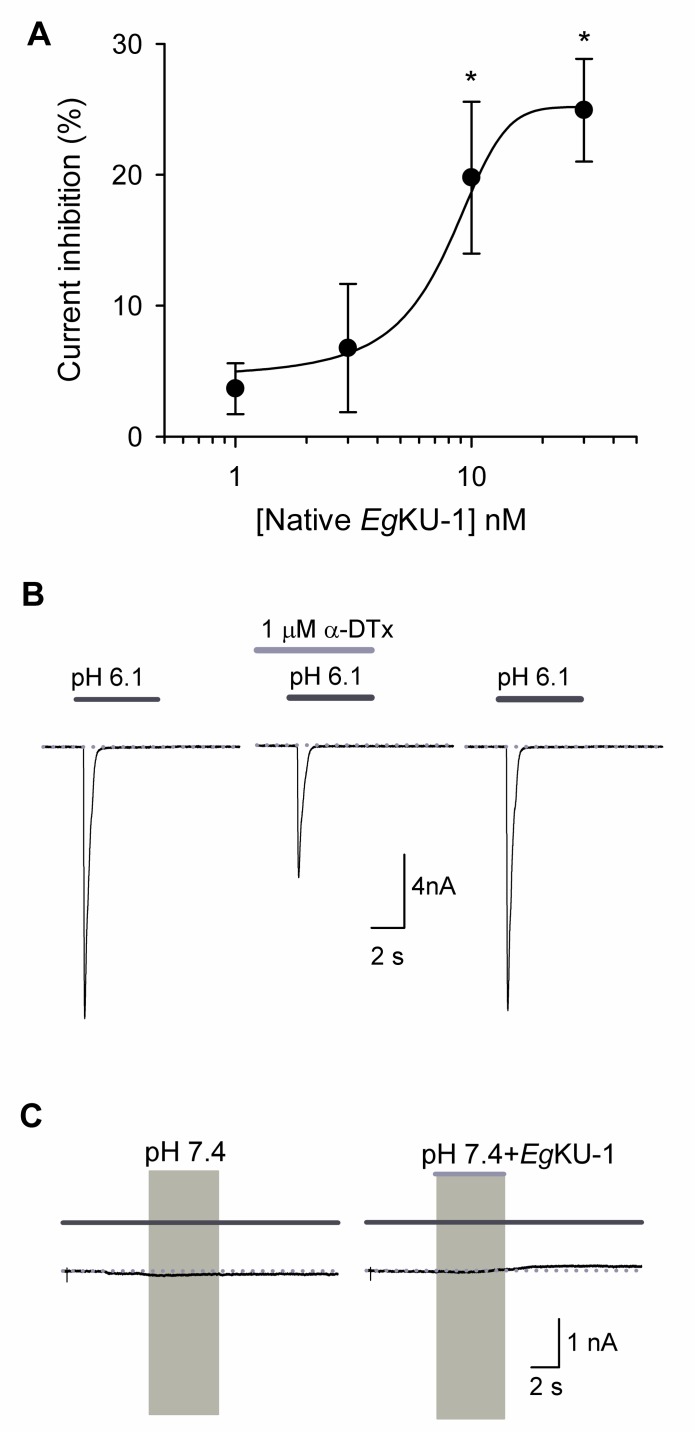
Concentration-response analysis of native *Eg*KU-1 on ASIC currents from DRG neurons. (A) Analysis of native *Eg*KU-1 inhibitory effect on the ASIC current amplitude (n = 26). The black line shows the best fit to the dose-response equation, from which the IC_50_ was calculated (7.8 ± 0.7 nM). The data correspond to the mean ± standard error (n ≥ 6 in all cases, except for 1 nM in which n = 4). The asterisks indicate Student’s *t*-test significance with respect to the effect in the absence of inhibitor (P ≤ 0.05). (B) and (C) correspond to positive and negative controls, respectively. (B) Representative traces showing the acid (pH 6.1, 5 s) activated current under control conditions (left), after sustained (25 s) perfusion of α-DTX (center), and after 1 min washout (right). α-DTX (1 μM; n = 6) significantly decreased the current amplitude (44.5 ± 7.0%; P = 0.045). (C) The application of *Eg*KU-1 in extracellular solution, without any pH change, had no effect.

Taking into account the recently described activity of α-DTX on ASIC currents in DRG neurons [22], we also analyzed the effect of *Eg*KU-1 and *Eg*KU-4 on pH-dependent Na^+^ currents. The sustained application of both *Eg*KUs blocked the ASIC currents elicited by a pH change from 7.4 to 6.1. The blocking effect was on the peak amplitude (*I*_peak_) and no significant effect was observed on the desensitization time course (τ_des_). The effect on the current amplitude was fully reversible after 1 min washing (Fig 7A–7C). We similarly analyzed the effect of *Eg*KU-3 and *Eg*KU-8: *Eg*KU-3 (n = 8) produced a slight but significant decrement of the *I*_peak_ (6.4 ± 3.0%; P = 0.02), whereas *Eg*KU-8 (n = 7) had no effect (6.2 ± 5.1%; P = 0.11) (Fig 7D–7E). Finally, we studied the concentration-response relationship for native *Eg*KU-1 (Fig 8A); the estimated IC_50_ value (about 8 nM) was 25-fold lower than the one determined for K_v_ (about 200 nM), suggesting higher selectivity of *Eg*KU-1 for ASICs than K_v_.

### The *Eg*KUs may be detected in metacestode and adult worm secretions

**Fig 9:**
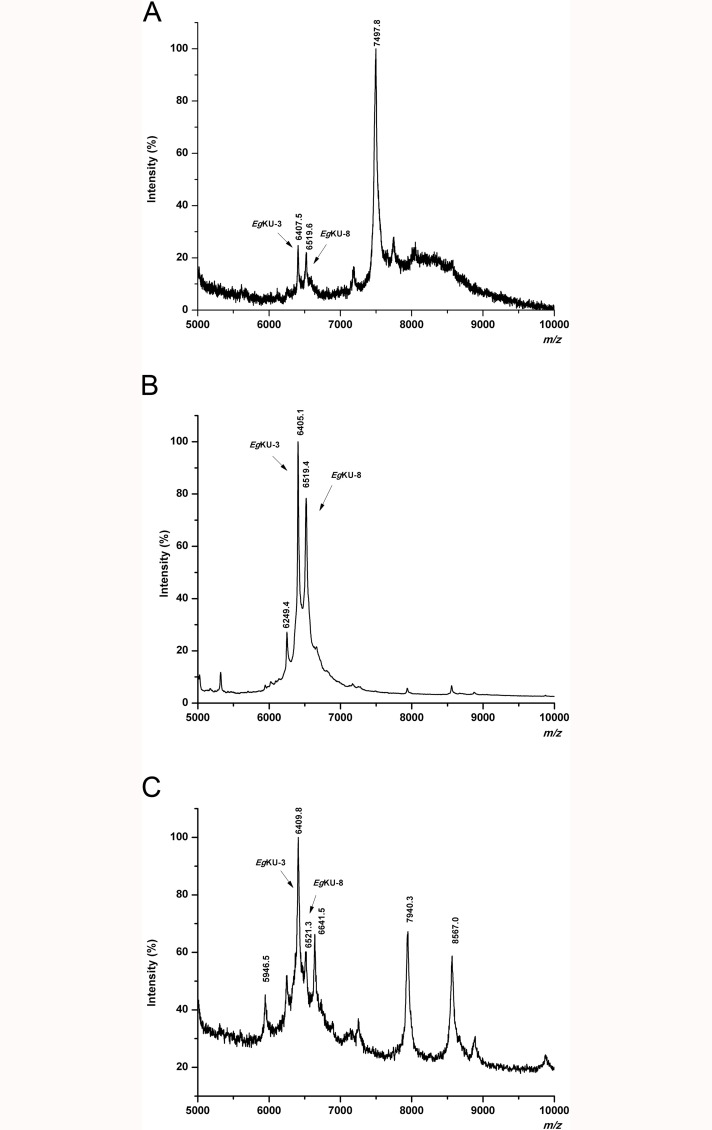
Detection of *Eg*KU-3 and *Eg*KU-8 in parasite secretions. Analysis by MALDI-TOF MS of hydatid fluid from a bovine cyst (A), as well as of chymotrypsin-affinity purified fractions from the same sample (B) and from the supernatant of cultured immature adults (C). Signals whose m/z values could derive from the *Eg*KUs are indicated (MH^+^ predicted for mature *Eg*KU-3 and *Eg*KU-8 are: 6406.8 and 6520.9, respectively). Note that the signals putatively corresponding to the *Eg*KUs are significantly enriched in the eluate from the affinity matrix. The identity of *Eg*KU-3 and *Eg*KU-8 purified from cyst fluid was subsequently confirmed by peptide mass fingerprinting (see S1 Dataset and the text for further details), as previously described [3].

**Fig 10:**
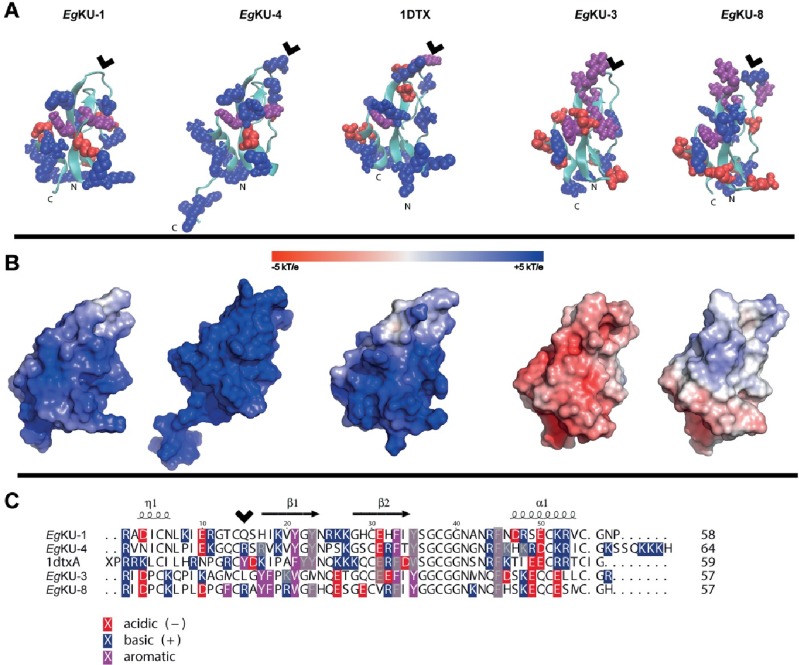
Structural analyses of *Eg*KU-1/*Eg*KU-4 and *Eg*KU-3/*Eg*KU-8. (A) Cartoon representation of structural models from the *Eg*KUs and the crystal structure of α-DTX (1DTX) featuring solvent-accessible (> 40 Å^2^) aromatic (purple), acid (red) and basic (blue) residues. N and C terminal ends are labeled. Note the presence of patches of basic amino acids with close aromatic residues in the models of α-DTX, *Eg*KU-1 and *Eg*KU-4. (B) Molecular surface electrostatic representations of the same proteins in the same orientation, highlighting global differences in charge distribution; scale represents charge from positive blue to negative red. (C) Sequence alignment produced with TEXshade [82] and hand-edited, featuring aromatic, acid and basic residues; those with solvent-accessibilities < 40 Å^2^ are grey shaded. Note that structurally equivalent positions in the *Eg*KUs and α-DTX are shifted two residues in the primary sequence. The P1 site of serine peptidase inhibitors, located at the center of the antipeptidase loop, is indicated with arrowheads in (A) and (C).

In our previous study, using mass spectrometry analysis, we showed that members of the Kunitz family, notably *Eg*KU-3 and *Eg*KU-8, would be present in protoscolex secretions from untreated and pepsin/H^+^-treated larval worms [3]. To further approach the question of whether Kunitz inhibitors are secreted to the parasite-host interface, we similarly analyzed hydatid fluid and adult worm secretions. Fig 9A shows a representative MALDI-TOF MS profile (5000–10000 Da) of hydatid fluid from bovine cysts. Peaks of m/z 6407.5 and 6519.6, matching the predicted MH^+^ value for *Eg*KU-3 (6406.4 Da) and *Eg*KU-8 (6520.4 Da), respectively, were observed. Furthermore, the intensity of the signals putatively corresponding to both *Eg*KUs was substantially increased in the chymotrypsin A-affinity purified fraction from the same sample (Fig 9B). In addition, the MS profile of an analogous fraction from the supernatant of *in vitro* cultured immature adults (Fig 9C) also showed peaks matching the predicted MH^+^ value for *Eg*KU-3 and *Eg*KU-8 (m/z of 6409.8 and 6521.3, respectively). Peptide mass fingerprinting of the components purified from cyst fluid allowed the detection of signals that could be assigned to tryptic peptides of these *Eg*KUs. In particular, we detected signals with m/z values 1074.54 and 1491.63 that corresponded to *Eg*KU-8 sequences 7LPLDPGFCR15 and 21WGFHQESGECVR32 with S-carboxymethylated cysteines (theoretical m/z values 1074.53 and 1491.64, respectively; see Fig 10C). In addition, a signal corresponding to *Eg*KU-3 sequence 49–57 was detected in the same spectrum. MS/MS analysis of the corresponding ions further corroborated the amino acidic sequences (S1 Dataset). Note that, although lower than towards trypsins, the affinity of *Eg*KU-8 towards chymotrypsin A (*K*_I_^*^ 10^−9^ M; Table 2) was high enough to allow its purification from the secretions. In contrast, this approach is not suitable to purify *Eg*KU-1 and *Eg*KU-4 that have no affinity towards chymotrypsin; thus, even if they had been present in the original sample, we would not have detected them.

## Discussion

In the present study, we described the functional characterization of eight members of the *E*. *granulosus* family of secreted Kunitz inhibitors (*Eg*KUs). Using recombinant forms of *Eg*KU-1-*Eg*KU-8 and native *Eg*KU-1, we demonstrated that six *Eg*KUs behave as high affinity inhibitors of either chymotrypsin (*Eg*KU-2 and *Eg*KU-3) or trypsin (*Eg*KU-5-*Eg*KU-8), whereas the close paralogs *Eg*KU-1/*Eg*KU-4 act as cation channel blockers (of K_v_ as well as ASICs), while showing either null (*Eg*KU-1, our previous study [3]) or marginal (*Eg*KU-4) serine peptidase inhibition activity. This degree of functional diversity, commonly observed in animal venoms, had not been previously described for Kunitz inhibitors present in parasite secretions.

Regarding serine peptidase inhibition, detailed kinetic studies showed that the interaction of *Eg*KU-3 with chymotrypsins mimics the one of the close paralog *Eg*KU-8 with trypsins: it is slow, of very high affinity and involves two steps. *Eg*KU-3 strongly inhibited isoforms A and B of chymotrypsin with *K*_I_^*^ in the 10^−11^ M range. Notably, according to MEROPS, dogs have two chymotrypsins B (with > 95% identity, encoded by CTRB1 and CTRB2 genes) and lack chymotrypsin A. The values of *K*_I_^*^ are among the smaller registered for chymotrypsin inhibitors. In fact, only two peptides have been reported to have similar affinity, both towards bovine chymotrypsin A (see S01.001 in MEROPS) [25,26]. No high affinity inhibitors of chymotrypsin B have been described so far, most likely because very few studies have been carried out with this isoform (see S01.152 in MEROPS). Therefore, *Eg*KU-3 appears as an interesting titration reagent for chymotrypsins A and B, especially for the latter because adequate titration reagents are currently unavailable. The stability of the *Eg*KU-3-chymotrypsin A complex is similar to that of *Eg*KU-8 complexes with trypsins, with *k*_2_/*K*_I_, the apparent second order rate constant for complex formation (*k*_on_), in the 10^6^ M^-1^ s^-1^ range; and the dissociation rate constant (*k*_-2_) in the 10^−4^ s^-1^ range (Table 3 and our previous study, [3]). These values of *k*_2_/*K*_I_ are in good agreement with reports for other members of the family, including BPTI with bovine trypsin [27], whereas those of *k*_-2_ are several orders faster than the one reported for BPTI (10^−8^ s^-1^ with bovine trypsin [27]).

The activity of *Eg*KU-3 as a strong tight-binding inhibitor of chymotrypsins and a less potent inhibitor of pancreatic elastase, as well as its lack of activity towards trypsin (Table 2) are consistent with the presence of a Leu in P1. In turn, similar to BPTI (Lys in P1), *Eg*KU-8 (Arg in P1) strongly inhibits trypsins, less potently chymotrypsin A and does not inhibit elastase (Table 2). Notably, neither *Eg*KU-8 nor BPTI inhibit chymotrypsin B. The antipeptidase loops of *Eg*KU-3 and *Eg*KU-8 differ by 50% (the corresponding mature polypeptides differ by only 37%); differences are mainly on the P side of the loop (residues 10 to 15), and involve some non-conservative substitutions (notably Asp10 instead of Lys10 in P6; see Fig 10C). Because the loop contributes to the inhibition specificity primarily determined by the P1 site, some of these residues are likely involved in the interaction with chymotrypsin B. In any case, consistent with the fact that isoforms A and B have similar affinities towards substrates with Phe as P1 residue [28], the values of *K*_*M*_ for the substrate we used were of the same order.

Regarding cation channel inhibition, patch-clamp studies carried out on rat DRG neurons showed that *Eg*KU-1 and *Eg*KU-4 block voltage-activated potassium currents. The effect was voltage-dependent and, as described for dendrotoxins, it was not totally reversible (Fig 3; [29]). The detailed characterization of *Eg*KU-1 activity on isolated K^+^ currents indicated that it preferentially blocks IK_DR_, as compared to IK_A_. This behavior differs from the one of α–DTX and resembles the one of δ–DTX [29]. In any case, the IC_50_ determined for *Eg*KU-1 was two orders of magnitude higher than those of dendrotoxins assayed on DRG neurons (10^−7^ *versus* 10^−9^ M [29]). This could be due, at least in part, to the fact that, although *Eg*KU-1 shares with dendrotoxins several residues that would participate in channel interaction (notably Leu7, Lys26 and Lys27), it lacks the Lys5 that has been described as a primary determinant of activity ([21]; this is also the case for *Eg*KU-4 that shares Leu7 and Lys27 with α–DTX and *Eg*KU-1; see Fig 10C). Because we studied the effect of the *Eg*KUs on total K_v_ currents of primary cultures, we cannot comment on their activity over specific K_v_. Nevertheless, our results indicate that *Eg*KU-1 could be more active over some K_v_ than others. Indeed, the dose-response curve does not start from zero (Fig 6), as if a specific K_v_ was highly blocked at low concentrations of the inhibitor.

*Eg*KU-1 and *Eg*KU-4 also showed a potent dose-dependent blocking effect on the ASIC currents in DRG neurons, which was totally reversible after one minute washing (Fig 7). These neurons express at least two subpopulations of transient ASIC currents as judged by their inactivation constants [30]. One of them derives from channels of ASIC1a, ASIC1b and ASIC3 subunits; the other from channels of ASIC2a subunits (reviewed by [31]), which are the least expressed in DRG [32]. Although our experimental setup does not allow us to conclude which channels are sensitive to the *Eg*KUs, the dispersion of the values in the dose-response study with native *Eg*KU-1 (Fig 8A) points to some variability of the blocking effect among different cells, suggesting that the effect could be stronger for some channel(s). *Eg*KU-1 could thus mimic the performance of other peptide blockers of ASICs, such as APETx2 (reviewed by [31]).

We recently reported that α-DTX, the well-known blocker of voltage activated K^+^ channels, also inhibits ASIC currents in rat DRG, although with significantly less potency than K_v_ (IC_50_ ~ 10^−7^ M [22] *versus* 10^−9^ M [29], respectively; see also Figs 4 and 8B). This result indicates that the Kunitz domain is yet another structural scaffold for ASIC-blocking polypeptides. Interestingly, an exposed basic-aromatic cluster identified in structurally different ASIC blocking peptides [33] was also found to be present in the structure of α-DTX [22]. Notably, this feature is observable towards one side of the model structures of *Eg*KU-1 and *Eg*KU-4 and not in those of *Eg*KU-3/*Eg*KU-8 (Fig 10A). In any case, functionally distinct *Eg*KUs differ mainly in surface charge distribution (Fig 10B). The relatively low selectivity of Kunitz inhibitors towards cation channels contrasts with the high specificity of their interaction with serine peptidases. Not surprisingly, structure-activity analyses focused at identifying the “channel-blocking site” of Kunitz proteins have usually highlighted regions on their surface involved in channel interaction but not a defined structural motif comparable to the antipeptidase loop involved in serine peptidase interaction. Furthermore, key residues for channel blockade are frequently located in the N- or C-terminal extensions of the Kunitz domain [17,34]

**Fig 11:**
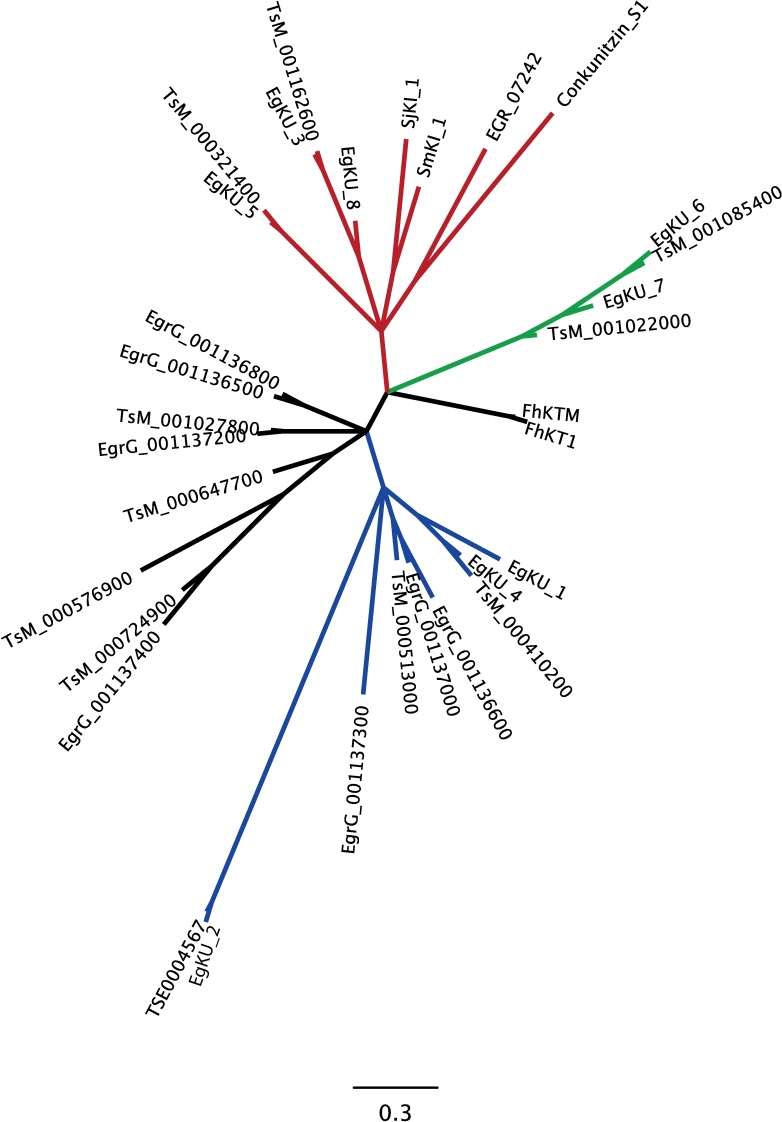
Expanded view of the *E*. *granulosus* Kunitz family. Unrooted phylogenetic tree highlighting sequence groupings within the family that roughly correlate with functional features described in the main text. Of outmost notice are sub-clades which include pairs such as the serine peptidase inhibitors *Eg*KU-3/*Eg*KU-8 (red clade) and *Eg*KU-6/*Eg*KU-7 (green clade); and the channel blockers *Eg*KU-1/*Eg*KU-4 (blue clade). Note that the sequences from *T*. *solium* pair with their close *E*. *granulosus* paralogs. Interestingly, the serine peptidase inhibitors SjKI-1 [37], SmKI-1 [38] and EGR_07242 (EgKI-2 in [36]) group in the red clade. The sequences from *F*. *hepatica* (FhKTM [42] and FhKT1 [43]) define a basal, separate clade that could reflect functional diversity (cysteine peptidase inhibition; [43]). The long branch of *Eg*KU-2 (and its putative *T*. *solium* ortholog) may reflect either a basal position of the protein (ancient/extreme sequence divergence), an accelerated evolution (*e*.*g*. through positive selection) or even relaxed selective pressures resulting in high tolerance to mutation accumulation. Data are insufficient to distinguish between such alternative scenarios. EgrG_001136500, in a black clade to the left, was also found to be a potent serine peptidase inhibitor (EgKI-1 in [36]). The position of the mollusk sequence (Conkunitzin S1), which was characterized as a channel blocker [14], is probably derived from the fact that, similar to EGR_07242, it lacks Cys14. This artifact is to be expected in short sequences. Bottom scale bar denotes average substitutions per site.

The availability of the *E*. *granulosus* genome [5,35] has allowed us to identify genes coding for at least eight additional monodomain Kunitz proteins with the same molecular architecture as *Eg*KU-1–*Eg*KU-8, *i*. *e*. a signal peptide followed by a single Kunitz domain. Similar to the rest of the family, the newly-identified members are diverse and include several pairs of close paralogs, consistent with an accelerated evolution of the family. Fig 11 shows an unrooted phylogenetic tree of the Kunitz domains from the sixteen *Eg*KUs together with eleven close paralogs from *T*. *solium* and five from functionally characterized monodomain Kunitz proteins from Lophotrochozoa, including four from trematodes. A true phylogenetic tree is not intended, as the signal might be blurred by homoplasy. Rather, the tree is aimed to mirror functional groupings of the sequences in an approximate evolutionary context. Not surprisingly, the sequences from *T*. *solium* pair with their close *E*. *granulosus* paralogs. The groupings roughly correlate with functional features, whereas *Eg*KU-2 (and a putative *T*. *solium* ortholog) appears very distant from the rest. The red sub-clade includes several serine peptidase inhibitors: in addition to *Eg*KU-3/*Eg*KU-8 and *Eg*KU-5, EGR_07242 (EgKI-2 in [36]) and the schistosome proteins SjKI-1 [37] and SmKI-1 [38]. EGR_07242 (Arg in P1) was recently found to inhibit trypsin, although with relatively low affinity (*K*_I_ ~ 10^−9^ M; [36]), probably due to the lack of Cys14, *i*. *e*. the one forming the disulphide bond that stabilizes the antipeptidase loop. SjKI-1 and SmKI-1 (both with Arg in P1) also inhibit trypsin with IC_50_ in the 10^−10^ and 10^−8^ M range, respectively [37,38]. The green sub-clade appears to group a different set of serine peptidase inhibitors (*Eg*KU-6/*Eg*KU-7 and closely related proteins from *T*. *solium*). In turn, the blue sub-clade includes the channel blockers *Eg*KU-1/*Eg*KU-4 together with another pair of close *E*. *granulosus* paralogs (EgrG001136600/EgrG001137000), and two *T*. *solium* proteins (TsM_000410200 and TsM_000513000). Although it is difficult to predict their function without further data, these proteins could also act as channel blockers because, similar to *Eg*KU-1/*Eg*KU-4, they feature the conserved Leu7 and a positively charged β–turn that form the K_v_-blocking site of α-DTX and related toxins [34,39,40,41]. As to the other groupings, EgrG_001136500 (Leu in P1) was recently found to be a potent inhibitor of neutrophil elastase (*K*_I_ ~ 10^−11^ M) and cathepsin G (*K*_I_ ~ 10^−10^ M) and, interestingly, to reduce neutrophil infiltration in a local inflammation model (EgKI-2 in [36]); thus, its close paralog (EgrG_00113800; Arg in P1) could also be a serine peptidase inhibitor. Finally, the sequences from *Fasciola hepatica* (FhKTM [42] and FhKT1 [43], both with Leu in P1, whose Kunitz domains differ in 3/51 amino acids) define a basal, separate sub-clade, that could also reflect functional diversity: FhKTM was found to be a marginal inhibitor of trypsin with virtually no effect over chymotrypsin [42] but, notably, FhKT1 was recently characterized as an inhibitor of cysteine peptidases, including the major parasite cathepsin L secreted peptidases and related human peptidases [43]).

Another interesting finding of our work refers to the demonstration of the presence of some *Eg*KUs (notably, *Eg*KU-3 and *Eg*KU-8) in cyst fluid (from bovine cysts) and secretions from immature adult worms, which complement our previous results with secretions from protoscoleces and pepsin/H^+^-treated protoscoleces [3]. *Eg*KU-8 was also detected by proteomic analyses in fertile cyst fluids from ovine and human infections, but not in infertile cysts from infected cattle [44]. Members of the Kunitz family could thus be secreted to the *E*. *granulosus*-dog interface not only at the initial stages of infection (as indicated by their presence in larval worm secretions) but also at late stages, and contribute to the establishment and persistence of dog echinococcosis. In turn, their presence in cyst fluid would point to a role at the onset of infection in dogs and/or during the chronic stage of infection in intermediate hosts.

In addition, available RNASeq data [5,35] indicate that members of the family are expressed in all the analyzed stages (immature adult, activated oncosphere, cyst, protoscolex, pepsin/H^+^-treated protoscolex); interestingly, most *Eg*KUs are expressed in adults. Furthermore, several of them appear up-regulated in this stage, notably, *Eg*KU-3, *Eg*KU-7, *Eg*KU-8, and also EGR_07242 and EgrG_0001137000 [35]. In addition, the orthologs of *Eg*KU-1 and *Eg*KU-4 were found to be highly up-regulated in *E*. *multilocularis* gravid adults (as compared to non-gravid worms) [5], whereas EgrG_001136500 and a close paralog (EmuJ_001136900) were among the transcripts with higher expression in oncospheres from *E*. *granulosus* [35] and *E*. *multilocularis* [45], respectively. Together with our results highlighting the presence of *Eg*KUs in parasite secretions, these data support the concept that Kunitz proteins are involved in parasite interaction with definitive and intermediate hosts, and indicate that specific members of the family would be engaged in particular moments of the life-cycle.

Given the activity profile of *Eg*KU-1-*Eg*KU-8 and the fact that they are mostly expressed and secreted by larval and adult worms [3,35], we can speculate about their potential counterparts in the dog duodenum. The apical end of the scolex contains a gland (the rostellar gland) whose secretion is believed to play a key role in host-parasite cross-talk, due to the very intimate contact of the scolex with the mucosa (reviewed by [2]). Interestingly, seminal studies demonstrated that the rostellar gland secretion is cystine-rich [46]; the gland could thus be the site of synthesis and concentration of the *Eg*KUs. Pancreatic enzymes appear as clear targets of the *Eg*KUs acting as serine peptidase inhibitors: those present in cyst fluid could initially protect the larval worms from digestion; whereas those secreted could protect the scolex (whose glycocalix is thin) after the parasite has attached to the mucosa. The *Eg*KUs could also inhibit other serine peptidases, such as those secreted by immune cells (see for example [47]) and membrane peptidases from epithelial cells (see for example [48,49]); in turn, this effect could prevent the activation of proteinase-activated receptors (PARs; [50]). As to putative targets of EgKU-1/EgKU-4, very little is known about the expression and functional properties of K_v_ and ASICs in the gut. In any case, both types of channels participate in the physiology of epithelial cells and afferent neurons [51,52,53]. In addition, K_v_ and ASICs are involved in the activation and maturation of dendritic cells and macrophages. In particular, K_v_1.3 and K_v_1.5 modulate the Ca^2+^-dependent functions of these cells and their blockade down-regulates their activation [54]. Regarding ASICs, dendritic cells express ASIC1, ASIC2 and ASIC3, and extracellular acidosis induces currents that are blocked by ASIC inhibitors. In addition, acidosis triggers the activation of dendritic cells and macrophages and ASIC inhibitors block these effects [54,55]. Taking into account that extracellular acidosis is a hallmark of inflammation, the blockade of ASICs may be crucial to weaken the induction of innate immunity and to favor the development of a chronic infection. In this context, it is pertinent to mention that excretory-secretory (E/S) products from *E*. *granulosus* adults have recently been found to impair dendritic cell function and induce the development of regulatory T cells [56]. Furthermore, a similar result was observed with the Kunitz protein FhKTM from the trematode *F*. *hepatica*, which is known to be present in parasite E/S products [57].As already mentioned, FhKTM showed a marginal serine peptidase inhibitory activity [42] but, notably, the very closely related FhKT1 (> 90% overall identity with FhKTM) was recently found to inhibit cysteine peptidases [43].

In our previous study, we included data from an extensive survey of platyhelminth ESTs available at the time, indicating that the expression of families of monodomain Kunitz proteins would be a distinctive trait of cestodes [3]. Genomic and transcriptomic data currently available (accessible from WormBase ParaSite: http://parasite.wormbase.org/; [58]) confirm and extend these initial observations, in particular, for the other medically important cestodes, *E*. *multilocularis* and *T*. *solium*, and indicate that this family is expanded in cestodes. Putative orthologs of virtually all the *Eg*KUs are present in the *E*. *multilocularis* genome, and several are also predicted for *T*. *solium* (Fig 11). In addition, recent genome-wide analyses of E/S proteins showed that the Kunitz domain is either the most (in *E*. *multilocularis*, 17/673 predicted E/S proteins; [59]) or the third most (in *T*. *solium*, 14/838 predicted E/S proteins; [60]) represented domain in the predicted secretome of these parasites, whereas manual inspection of the putative secreted Kunitz proteins indicates that a majority of them contain a single Kunitz domain. In contrast, parasitic trematodes express only a few monodomain Kunitz inhibitors (see for example [61]).

The secretion of monodomain Kunitz proteins thus appears to be a strategy evolved by cestodes to block, through high affinity interactions, the function of host proteins (either serine peptidases or cation channels) and contribute to the establishment and persistence of infection. The putative immunomodulatory role of these molecules opens the way to further studies of their involvement in immunoevasion, acting as single molecules as well as synergistically. From a more general perspective, the data confirm that multigene families of Kunitz inhibitors from parasite secretions and animal venoms display a similar functional diversity. As we had previously mentioned, because the genes coding for parasite secretions and predator toxins arise from an arms race between different organisms, it is interesting to consider that both sets of molecules display analogous evolutionary patterns. Finally, the strong target specificity of some of these molecules makes them uniquely suited as tools for the characterization of biological processes as well as for the development of pharmaceuticals.

## Materials and methods

### Preparation of *Eg*KUs

Native *Eg*KU-1 and *Eg*KU-8 were purified to homogeneity from a protoscolex lysate by cation exchange followed by reverse-phase chromatography, as previously described [3].

*Eg*KU-1–*Eg*KU-8 [3] were overexpressed as amino-terminal His6-tagged fusion proteins in *Escherichia coli* strain BL21(DE3) using pET28a recombinant plasmids prepared according to standard procedures. The expression constructs included the His6 leader sequence followed by the cDNA sequence encoding the corresponding predicted full-length mature *Eg*KU. These were amplified from *E*. *granulosus* pepsin/H^+^-activated protoscolex cDNA [62] using *Vent* DNA polymerase (New England Biolabs) and specific primers containing restriction sites to allow directional cloning into the pET28a vector. *Bam*HI and *Hind*III sites were used except for *Eg*KU-6 and *Eg*KU-7 whose coding sequences are cleaved by *Bam*HI; *Eco*RI was used instead. The integrity of the expression constructs was checked by sequencing. The His6-tagged *Eg*KU fusion proteins were expressed in transformed *E*. *coli* grown in LB containing 10 mg/L of kanamycin and induced at 37°C with 0.1 mM isopropyl thiogalactopyranoside. Induction of expression was at late-log-phase (A_600_ 0.6–1.0) during 4 h, in the case of the pairs of paralogs *Eg*KU-1/*Eg*KU-4 and *Eg*KU-3/*Eg*KU-8 that yielded good amounts of soluble recombinant peptides [63]. Expression of *Eg*KU-2, *Eg*KU-5 and the paralogs *Eg*KU-6/*Eg*KU-7, whose recombinants are recovered mostly as inclusion bodies [63], was induced earlier (A_600_ 0.2–0.3) to maximize the yield of the soluble proteins. In all cases, the induced cells were harvested by centrifugation, the pellet was suspended in “lysis” buffer (50 mM NaH_2_PO_4_, 300 mM NaCl, 10 mM imidazole), and the cells were lyzed by sonication. The lysates were centrifuged (20,000 *g* for 30 min at 4°C) and the supernatants used to purify the His-tagged fusion proteins using a Ni^2+^-charged affinity matrix (Ni-NTA, Invitrogen), following the manufacturer’s instructions. The soluble fraction of the bacterial lysates was loaded onto the column equilibrated with lysis buffer, washed with equilibration buffer including 30 mM imidazole, and the recombinant *Eg*KUs eluted with the same buffer containing 250 mM imidazole, that was subsequently dialyzed.

The purity of the *Eg*KUs was checked by SDS-PAGE analysis and the protein concentration was determined with the bicinchoninic acid reagent (BCA, Pierce, USA) using bovine serum albumin as standard, or by A_280_. The quality of the recombinants was further controlled by: i) confirming the presence of three disulphide bonds (*i*. *e*. that the proteins were fully oxidized) through determination of the molecular masses of the *Eg*KUs and their reduced and alkylated derivatives by MALDI-TOF MS, as described by Calvete [64] (S2 Fig); ii) checking that recombinant *Eg*KU-8 reproduced the performance of the native inhibitor towards bovine trypsin and thus, that the recombinant was properly folded and the N-terminal extension contributed by the expression vector did not interfere with enzyme interaction (*K*_i_^*^ was 50 ± 10 pM for recombinant *Eg*KU-8 and 60 ± 13 pM for the native inhibitor [3]); iii) during this study, we also verified that recombinant *Eg*KU-1 reproduced reasonably well the performance of the native inhibitor acting on K_v_ and ASIC currents from DRG neurons (Figs 3 and 7, respectively). Usual yields of the *Eg*KUs recovered as soluble recombinants and used for activity assays were as follows: ~5 mg/L of culture for *Eg*KU-1/*Eg*KU-4 and *Eg*KU-3/*Eg*KU-8; ~300 μg/L for *Eg*KU-7 (~30% of the total); and ~5 μg/L for *Eg*KU-2, *Eg*KU-5 and *Eg*KU-6 (~5% of the total). The purity of the proteins used for detailed activity studies (*Eg*KU-1, *Eg*KU-3, *Eg*KU-4 and *Eg*KU-8) was always > 95%.

### Assays of peptidase inhibition by *Eg*KUs

The inhibitory activity of recombinant *Eg*KU-1–*Eg*KU-8 was tested against bovine trypsin (EC 3.4.21.4), bovine and canine chymotrypsins (EC 3.4.21.1), and porcine elastase (3.4.21.36), essentially as previously described [3]. Bovine enzymes and porcine elastase were obtained from Sigma-Aldrich, whereas the canine peptidase was purified from the pancreas of a dog that had passed away due to an accidental cause, following the procedure of Waritani *et al*. [65]. According to MEROPS, dogs have two chymotrypsins B (with > 95% identity, encoded by CTRB1 and CTRB2 genes); the fraction we isolated from dog pancreas most likely contained a mixture of both enzymes.The following peptidases were thus assayed (MEROPS—http://merops.sanger.ac.uk—identifiers are indicated in brackets; [9]): from *Bos taurus*, chymotrypsin A (S01.001) and trypsin 1 (cationic, S01.151); from *Sus scrofa*, elastase (S01.153); from *Canis familiaris*, chymotrypsin B (S01.152)).

Prior to inhibition studies, proteolytic activity in enzyme preparations was determined with fluorogenic substrates using initial steady-state rate conditions at 37°C and pH 8.0. Assays (200 μl) were performed in black 96-well microplates (Costar, Corning Life Sciences). Enzymes and substrates were dissolved in 50 mM Tris-HCl, pH 8.0 containing 0.01% Triton X-100 (v/v), and reactions were initiated by the addition of enzyme. The changes in fluorescence intensity, corresponding to the formation of the hydrolysis product 7-amino-4-methylcoumarin (AMC), were registered at excitation and emission wavelengths of 390 and 460 nm, respectively, with a microplate fluorescence reader (FLUOstar* OPTIMA, BMG Labtechnologies). For trypsin activity, the artificial substrate N-*t*-BOC-Ile-Glu-Gly-Arg-AMC was used; for chymotrypsin, Suc-Ala-Ala-Pro-Phe-AMC; and for elastase, Suc-Ala-Ala-Ala-AMC. Calibration curves using AMC were carried out in each experiment. Initial steady-state rates of substrate hydrolysis were calculated from the linear portion of product (AMC) *versus* time plots when less than 10% of substrate had been consumed. The substrates and AMC were also obtained from Sigma-Aldrich.

Protein concentrations of enzyme preparations were determined with the BCA reagent using bovine serum albumin as standard; and the active site concentration of trypsin and bovine chymotrypsin A by specific titration with the high affinity inhibitor BPTI. Initially, the active site concentration of canine chymotrypsin could not be estimated because, similar to bovine chymotrypsin B [66], it was not inhibited by BPTI. The enzyme was subsequently titrated with *Eg*KU-3 that inhibits chymotrypsins A and B with high affinity (see results).

The kinetic parameters for substrate and enzyme pairs were calculated from the non-linear fitting to the Michaelis-Menten equation. The values determined with the substrates specified above were: *K*_*M*_ = 85 ± 9 μM and k_Cat_ = 50 ± 6 s^-1^ for bovine trypsin; *K*_*M*_ = 30 ± 2 μM and k_Cat_ = 19 ± 2 s^-1^ for bovine chymotrypsin A; *K*_*M*_ = 39 ± 2 μM and k_Cat_ = 6 ± 1 s^-1^ for canine chymotrypsin B.

For inhibition studies, each of the enzymes was incubated with the purified recombinant *Eg*KUs for 15 min at 37°C prior to the addition of the appropriate fluorogenic substrate, to allow for the equilibration of the enzyme-inhibitor complexes. The substrate concentration (5 μM) was chosen so as to be well below the corresponding *K*_*M*_, as specified above.

To check whether the *Eg*KUs behaved as high affinity inhibitors, the purified recombinants were titrated against active-site titrated bovine trypsin and chymotrypsin, as described by Olson *et al*. [67].

### Peptidase inhibition studies with *Eg*KU-3 and *Eg*KU-4

The activities of *Eg*KU-3 and *Eg*KU-4 were further analyzed by characterizing the kinetics of enzyme inhibition, as previously described for *Eg*KU-8 [3]. All experiments were carried out at least two independent times. Within each experiment, measurements were performed in duplicates. The inhibition and rate constants reported are the average ± standard error of independent experiments.

#### Tight-binding kinetics

In order to determine the global inhibition constants (*K*_I_^*^) of the recombinant inhibitors towards the assayed serine peptidases, the initial steady-state rates of substrate hydrolysis in the presence of increasing concentrations of each inhibitor were measured after pre-incubation of the enzyme with inhibitor. The inhibition constants were calculated by nonlinear fitting to the Morrison equation for tight binding inhibitors [68,69]:

$$v_{i}=\frac{v}{2\left[\text{E}\right]}\left\{\left(\left[\text{E}\right]-\left[\text{I}\right]-K_{I\;app}^{*}\right)+\sqrt{{\left(\left[\text{I}\right]+K_{I\;app}^{*}-\left[\text{E}\right]\right)}^{2}+4K_{I\;app}^{*}\left[\text{E}\right]}\right\}$$ (1)

where *K*_I_^*^_app_ is the apparent global dissociation constant of the enzyme-inhibitor complex, *v*_*i*_ is the inhibited steady-state rate, *v* is the uninhibited rate, [I] is the total inhibitor concentration and [E] is the total enzyme concentration. The true inhibition constants, *K*_I_^*^, were corrected from *K*_I_^*^_app_ according to the Eq 2 for competitive inhibitors:

$$K_{\text{I}}^{*}=\frac{K_{I\;app}^{*}}{1+\frac{\left[\text{S}\right]}{K_{M}}}$$ (2)

#### Slow-binding kinetics

The decrease in the rate of product formation during the first minutes after mixing the enzyme with the inhibitor and substrate (5 μM) was studied for increasing inhibitor concentrations. Progress curves were analyzed using the Eq 3 [70] that describes the slow establishment of equilibrium between the enzyme and the inhibitor according to:

$$\text{P}=v_{i}t+\frac{\left(v_{0}-v_{i}\right)\left(1-e^{-k_{\text{obs}}t}\right)}{k_{\text{obs}}}$$ (3)

where P is the concentration of AMC produced by hydrolysis of the substrate, *v*_*o*_ is the initial rate, *v*_*i*_ is the rate once the enzyme-inhibitor equilibrium was reached, and *k*_obs_ represents the apparent first order rate constant. Computer fitting of progress curves estimated values for *v*_*o*_, *v*_*i*_ and *k*_obs_.

For Kunitz inhibitors that bind to the enzyme rapidly and reversibly forming an initial “loose” complex EI that isomerizes slowly to the final complex EI*, the reaction mechanism can be represented by Eq 4:

E+I⇌EI⇌EI* (4)

In this mechanism, the value of the apparent rate constant (*k*_obs_) is related to the kinetic constants of the second step, *k*_2_ and *k*_-2_, and to the equilibrium dissociation constant of the initial loose complex *K*_I_ (*K*_I_ = *k*_*-1*_*/k*_*1*_), by Eq 5 [24]:

$$k_{\text{obs}}=k_{-2}+\frac{k_{2}\left[\text{I}\right]}{\left[\text{I}\right]+K_{\text{I}}\left(1+\left[\text{S}\right]/K_{M}\right)}$$ (5)

In the case of *Eg*KU-3 that was found to follow the mechanism represented in Eq 4, the constants *k*_*2*_ and *K*_I_ were determined from plots of *k*_obs_ *versus* [I], by computer fitting to Eq 5. Because *k*_*-2*_ was too small to be accurately estimated from these plots, it was determined with Eq 6 [23] using data from situations where the ratio *v*_*i*_*/v*_*o*_ was higher than 0.05:

$$k_{-2}=k_{\text{obs}}\frac{v_{i}}{v_{0}}$$ (6)

The values of *k*_*-2*_, *k*_*2*_ and *K*_I_ thus determined allowed to corroborate the inhibition constant *K*_I_^*^, according to Eq 7:

$$K_{\text{I}}^{*}=K_{\text{I}}\frac{k_{-2}}{k_{2}+k_{-2}}$$ (7)

#### Data analysis

Computer fitting to non-linear equations was performed using the software Origin version 8 (OriginLab).

### Patch-clamp experiments with *Eg*KU-1 and *Eg*KU-4 on DRG neurons

The effect of *Eg*KU-1 and *Eg*KU-4 on voltage-gated (Na^+^ and K^+^) and ASIC currents was studied using the whole cell patch-clamp technique in primary cultured rat DRG neurons. The effect of *Eg*KU-3 and *Eg*KU-8 on voltage-gated K^+^ and ASIC currents was similarly analyzed. α-DTX (kindly donated by Dr. Carlos Cerveñansky from the Unidad de Bioquímica y Proteómica Analíticas, Institut Pasteur de Montevideo/Instituto de Investigaciones Biológicas Clemente Estable, Montevideo, Uruguay) and Bovine Serum Albumin (Sigma Chemicals) were used as positive and negative controls, respectively.

#### Ethics statement

The study was performed in strict accordance with the Guiding Principles from the Committee on Guide for the Care and Use of Laboratory Animals of the National Research Council of the National Academies of the United States of America and with the regulations of the General Law of Health and of Research in Health Matters of the Ministry of Health of Mexico and Technical Specifications for production, use and Care of laboratory animals (NOM-062-ZOO-1999). The animal protocol was reviewed and approved by the Institutional Animal Care and Use Committee (IACUC) of the Vice-rectory of Research and Postgraduate Studies of the Autonomous University of Puebla (BUAP/VIEP 2014–236, BUAP/VIEP 2015–273 and BUAP/VIEP 2016–265). All efforts were made to minimize animal suffering and to reduce the number of animals used. The animals were provided by the “Claude Bernard” animal facility of the Autonomous University of Puebla.

#### Cell culture

The DRG neurons were isolated from the vertebral column of Wistar rats at postnatal ages P5 to P9 without sex distinction, and cultured according to the procedure described by Salceda and coworkers [71]. In brief, the neurons were incubated (30 min at 37°C) in Leibovitz L15 medium (Invitrogen, USA) containing 1.25 mg/ml trypsin and 1.25 mg/ml collagenase (both from Sigma-Aldrich). After the enzymatic treatment, the ganglia were washed 3 times with sterile L15. Cells were mechanically dissociated and plated on 12-mm x 10-mm glass coverslips (Corning, USA), pretreated with poly-D-lysine (Sigma-Aldrich), which were placed onto 35-mm culture dishes (Corning). The isolated cells were allowed to settle and adhere to the coverslips during 4 to 8 h in a humid atmosphere (95% air and 5% CO_2_, at 37°C) using a CO_2_ water-jacketed incubator (Nuaire, USA). The plating medium was L15 supplemented with 15.7 mM NaHCO_3_ (Merck, Mexico), 10% fetal bovine serum, 2.5 μg/ml fungizone (both from Invitrogen), 100 U/mL penicillin (Lakeside, Mexico), and 15.8 mM HEPES (Sigma-Aldrich).

#### Electrophysiological recording

A coverslip with attached cells was transferred to a 500 μl perfusion chamber mounted on the stage of an inverted phase-contrast microscope (TMS, Nikon Co, Japan). Cells were bath-perfused with extracellular solution by means of a peristaltic pump (Masterflex, L/S Easy-Load II; Cole Parmer, USA). Current recording was carried out at room temperature (23–25°C) using an Axopatch 1D amplifier (Molecular Devices, USA). The cells selected for recording were refringent, they were not adhered to other cells, showed no neurite outgrowth, and had a round soma. Command-pulse generation and data sampling were controlled with the pClamp 9.2 software (Molecular Devices) using a 16-bit data-acquisition system (Digidata 1320, Molecular Devices). Signals were low-pass filtered at 5 kHz and digitized at 10 or 20 kHz, depending on the experiment. Patch pipettes were pulled from borosilicate glass capillaries (TW120-3; WPI, USA) using a Flaming-Brown electrode puller (P-80/-PC; Sutter Instruments, USA). They typically had a resistance of 1 to 3 MΩ when filled with intracellular solution. The series resistance was electronically compensated for by ≈ 80%. Seal and series resistance were continuously monitored to guarantee stable recording conditions.

#### Experimental protocols and data analysis

To study their effects on voltage-gated K^+^ and Na^+^ currents, the *Eg*KUs were ejected under pressure using a microinjector (Baby Bee, USA) from a micropipette positioned in the vicinity of the cell under recording. For the recording of total currents, the cells were perfused with an extracellular solution containing (mM): 140 NaCl, 5.4 KCl, 1.8 CaCl_2_, 1.2 MgCl_2_, 10 HEPES, adjusted to pH 7.4 with NaOH. For the recording of ASIC currents, the same solution was employed but HEPES was substituted by MES (10 mM) and pH was adjusted to 6.1. For these experiments, the patch pipettes were filled with an intracellular solution containing (mM): 10 NaCl, 125 KCl, 0.1 CaCl_2_, 10 EGTA, 5 HEPES, 1 NaGTP, 2 MgATP, adjusted to pH 7.2 with KOH.

Potassium transmembrane currents were elicited by a single-step voltage protocol applying a 120 ms pre-pulse to -100 mV before the 0 mV (800 ms) test pulse with an interval between sweeps of 8 s (holding potencial, V_h_ = -60 mV). For current-voltage (I/V) analyses, the total potassium current was obtained from a V_h_ of -45 mV through a series of voltage steps (from -65 to 55 mV, every 8 s) preceded by a conditioning step to -120 mV (200 ms). To record the sustained component of the current (IK_DR_), the conditioning step was set to -45 mV to inactivate the transient component of the current (IK_A_). Then IK_A_ was calculated by a trace by trace subtraction between the aforementioned protocols. For this group of experiments, the extracellular solution contained (mM): 10 KCl, 1.8 CaCl_2_, 1.2 MgCl_2_, 0.3 CdCl_2_, 130 choline chloride, 10 HEPES, adjusted to pH 7.4; and the intracellular solution (mM): 50 KCl, 60 choline chloride, 0.1 CaCl_2_, 40 KF, 10 EGTA, 5 HEPES, adjusted to pH 7.2.

The *Eg*KUs were always co-applied with the single-step voltage protocol. I/V analyses were carried out after the effect had stabilized. Two parameters were used to evaluate the effect: the maximum amplitude of the current and the amplitude of the current in the last 10 ms of the test pulse.

Sodium currents were evoked by a 40 ms depolarization step to a membrane potential of -10 mV from a V_h_ of -100 mV, with an interval between sweeps of 8 s. Three parameters were used to evaluate the effect of the *Eg*KUs: the maximum amplitude of the current (INa_max_), the time constant of the current inactivation (τ_h_), as derived from an exponential fit, and the ratio between the current amplitude at the end of the voltage pulse and INa_max_ (INa_end_/INa_max_), which gives an estimate of the probability for the channels not being inactivated at the end of the voltage pulse. Cells were bath-perfused with an extracellular solution containing (mM): 20 NaCl, 70 choline chloride, 1.8 CaCl_2_, 1 MgCl_2_, 10 HEPES, 45 tetraethylammonium chloride, 10 4-aminopyridine adjusted to pH 7.4. Pipettes were filled with the following intracellular solution (mM): 10 NaCl, 30 CsCl, 100 CsF, 5 HEPES, 8 EGTA, 10 tetraethylammonium chloride, adjusted to pH 7.2.

The ASIC currents were generated by a fast (about 100 ms) pH change from 7.4 to 6.1, by shifting one of the three outlets of a fast change perfusion system (SF-77B, Warner Inst, USA) while keeping the cell at a V_h_ of -60 mV (the extracellular solution of pH 6.1 contained MES, pK = 6.15, instead of HEPES, pK = 7.55). The pH was kept at 6.1 during 5 s. The time between pH changes was 1 min to guarantee that the ASIC current was completely recovered from desensitization. The transient receptor potential vanilloid 1 (TRPV1) antagonist capsazepine (10 μM) (Sigma-Aldrich) was added to the extracellular solution (pH 6.1) to prevent activation of the TRPV1 receptor present in DRG neurons. The ASIC currents were characterized by the maximum peak amplitude (*I*_peak_), the desensitization time-constant (τ_des_, determined by fitting the decay phase of the current with a single exponential function), and the amplitude at the steady state (*I*_end_) measured at the last 100 ms of the acid pulse. The *Eg*KUs were applied 20 s before and during the acid pulse (sustained application). At least two control responses were recorded before any experimental manipulation. The pH of the perfusion solution was checked not to be affected by addition of the inhibitors.

Concentration-response data were fitted with the function Y = min + (max-min)/[1+(x/EC_50_)^H^], where Y is the effect of the inhibitor, x is the concentration, max and min are the maximum and minimum effects, EC_50_ is the concentration at which 50% of the effect is obtained and H is the Hill coefficient. Experimental data are presented as the mean ± standard error. To define the statistical significance, a paired Student’s *t*-test was used and P ≤ 0.05 was considered as significant, when comparing the effects in the presence and absence of inhibitor.

### Analysis of *Eg*KUs in parasite secretions

Fresh hydatid fluid was recovered under aseptic conditions from individual fertile cysts of the G1 genotype (*E*. *granulosus sensu stricto*), present in the lungs of naturally infected bovines in Uruguay, and kept at -70°C. Cysts were collected during the routine work of local abattoirs in Montevideo. Cyst fluid was analyzed by MALDI-TOF MS using a Voyager DE-PRO spectrometer (Applied Biosystems). The sample was concentrated by vacuum drying, desalted using C18 reverse phase micro-columns (OMIX Pipette tips, Varian) and eluted with matrix solution (α-cyano-4-hydroxycinnamic acid in 0.2% trifluoroacetic acid in 50% v/v acetonitrile-H_2_O) directly on the MALDI sample plate.

An aliquot of adult worm *in vitro* secretions was kindly provided by MSc Noelia Morel; the culture was carried out in our department in the context of a project to develop a copro-ELISA kit for canine echinococcosis [72]. Worm secretions were prepared essentially as described by Casaravilla and coworkers [73]. In brief, adult worms were recovered from the intestine of dogs experimentally infected with *E*. *granulosus* (euthanized 30 days post-infection), washed with sterile phosphate-buffered saline (PBS) and cultured in RPMI containing 10^5^ UI/L penicillin, 100 mg/L streptomycin and 250 μg/L amphotericin B, at 37°C in 5% CO_2_.The supernatant was collected every 8 h for two days and kept at -70°C. An aliquot was clarified by centrifugation at 10,000 *g* and analyzed as described for cyst fluid.

*Eg*KU-3 and *Eg*KU-8 were affinity purified from hydatid fluid and worm secretions using chymotrypsin A agarose (Sigma-Aldrich, USA) and analyzed by MALDI-TOF MS (4800 MALDI TOF/TOF analyzer, ABi Sciex) to confirm their presence in the parasite secretions. The resin containing agarose-bound bovine chymotrypsin A (1.5 mg) was rehydrated and equilibrated with 10 mM Tris-HCl buffer pH 7.5. Cyst fluid (1 ml) was incubated in batch with the resin during 10 min at 20°C; the resin was then washed 3 times with 10 mM Tris-HCl pH 7.5, and the *Eg*KUs were eluted by incubation during 10 min with 25 μl of 0.2% trifluoroacetic acid. After centrifugation, 1 μl of the eluate was applied directly on the MALDI sample plate with 1 μl of the matrix solution (α-cyano-4-hydroxycinnamic acid in 0.1% trifluoroacetic acid in 60% v/v acetonitrile-H_2_O). Mass spectra were acquired in positive ion linear mode and externally calibrated using protein standards (Applied Biosystems). The *Eg*KUs were purified from adult worm secretions with the same protocol, using 3 ml of the sample previously concentrated by vacuum drying.

*Eg*KU-3 and *Eg*KU-8 purified from cyst fluid were characterized by peptide mass fingerprinting; the eluate was reduced and alkylated with iodoacetamide prior to treatment with trypsin (Sequencing-grade, Promega). The sequence of selected peptides was confirmed by collision-induced dissociation MS/MS experiments, as previously described [3].

### Structural modeling

The full-length mature sequences of *Eg*KU-1, *Eg*KU-3, *Eg*KU-4 and *Eg*KU-8 were used to compute structural models using the i-Tasser server [74]. The C-scores for all models were higher than 0 (*Eg*KU-1 = 0.93; *Eg*KU-3 = 1.23; *Eg*KU-4 = 0.05; *Eg*KU-8 = 1.13). Typical C-scores range from [–2, 5], with higher scores meaning more reliable models. α-DTX did not stand amongst the top 10 threading templates used by i-Tasser, which allowed direct comparisons with no circularities in the analyses. Electrostatic properties were calculated at pH 7.4 with the Adaptive Poisson Boltzmann Solver [75] for the best i-Tasser models as well as for the crystal structure of α-DTX (PDB access code 1DTX). Next, per-residue solvent-accessibility was computed with the “areaimol” program from the CCP4 suite [76]. Basic, acidic and aromatic residues with solvent accessibilities above 40 Å^2^ were displayed in van der Waals representation onto a cartoon backbone of the model, using VMD [77]. Electrostatic molecular surface representations were produced and rendered with PyMol (http://pymol.sourceforge.net).

### Phylogenetic analysis

The Kunitz domains from *Eg*KU-1-*Eg*KU-8 as well as from eight additional monodomain Kunitz proteins identified in the *E*. *granulosus* genome sequences (encoded by EgrG_001136500, EgrG_001136600, EgrG_001136800, EgrG_001137000, EgrG_001137200, EgrG_1137300 and EgrG_1137400 from the genome produced at the Wellcome Trust Sanger Centre [5]; and the protein EGR_07242, from the genome produced at the Chinese National Human Genome Center [35]) were multiply aligned with Mafft [78] (L-insi option). Eleven close paralogs (some of which are putative orthologs) of the *E*. *granulosus* monodomain Kunitz proteins identified within genomic and transcriptomic data from the *T*. *solium* genome project [5] (encoded by genes TsM_000321400, TsM_000410200, TsM_000513000, TsM_000576900, TsM_000647700, TsM_000724900, TsM_001022000, TsM_001027800, TsM_001085400, TsM_001162600 retrieved from GeneDB—http://www.genedb.org/Homepage/Tsolium; and the EST EL746785 retrieved as TSE0004567 from PartiGeneDB—http://www.compsysbio.org/partigene/) were added to this set. A group of five functionally annotated sequences from other Lophotrochozoa was also included: FhKTM (UniProt Q9TXD3; [42]) and FhKT1 [43], from *F*. *hepatica*; SjKI-1 (Sjp_0020270) from *S*. *japonicum* [37]; SmKI-1 (Smp_147730) from *S*. *mansoni* [38] and Conkunitzin-S1 (UniProt P0C1X2) from the mollusk *Conus striatus* [14]. The alignment of these 32 proteins was used as input for MrBayes [79] for a Bayesian phylogenetic reconstruction using the Poisson substitution model in a run of 1,000,000 generations, discarding the first 100,000 for summarizing results (mcmc ngen = 1000000; sump burnin = 100000; sumt burnin = 100000). The short sequence length of the Kunitz domain prevents robust and reliable identification of many branching events. Furthermore, saturation events are guaranteed to occur and may be difficult to pinpoint. The tree is one of the best we can get with current methods. In fact, maximum likelihood reconstruction with 1000 bootstraps provided poorer support for branching events. The final dendrogram was visualized and rendered in FigTree (http://tree.bio.ed.ac.uk/software/figtree). It should be noted that the proteins encoded by EgrG_001136500, EgrG_001136600, EgrG_001137000, EgrG_001137200, and EgrG_001137400 [5] correspond to EGR_08721, EGR_08720, EGR_08716, EGR_9006, and EGR_9007 [35], respectively.

## Supporting information

S1 Dataset Peptide mass fingerprinting of chymotrypsin affinity purified proteins from hydatid cyst fluid. (A) MALDI-TOF mass spectrum of peptides generated by tryptic digestion of cyst fluid proteins recovered after an affinity purification step using chymotrypsin A. The signals that can be assigned to peptides derived from *Eg*KU-8 or *Eg*KU-3 are indicated (refer to Fig 10C). The signal of m/z 1074.54 corresponds to *Eg*KU-8 sequence LPLDPGFcR (theoretical MH^+^ 1074.53); the signal of m/z 1164.52 to *Eg*KU-3 sequence EQcELLcGR (theoretical MH^+^ 1164.51); and the signal of m/z 1491.63 to *Eg*KU-8 sequence WGFHQESGEcVR (theoretical MH^+^ 1491.64); c indicates carbamidomethyl Cys. (B) MS/MS analysis of tryptic peptides from *Eg*KU-3 and *Eg*KU-8. The list of theoretical m/z values of fragment ions is shown for each sequence, and the ions detected in the MS/MS spectra are highlighted in bold. **a** and **b** ions correspond to N-terminal fragments, and **y** ions to C-terminal fragments, according to the accepted nomenclature [80,81].
(PDF)

S1 Fig Studies with *Eg*KU-1 and *Eg*KU-4 on isolated Na^+^ currents from DRG neurons. Representative traces showing that the sustained (25 s) perfusion of 100 nM recombinant *Eg*KU-1 (A) or *Eg*KU-4 (C) does not block voltage-activated sodium channels (Na_v_). (B) and (D) are the current-voltage relationships of the peak Na^+^ current from the traces in (A) and (C), respectively. The black traces correspond to control conditions and the gray ones after *Eg*KU perfusion.
(TIF)

S2 Fig MALDI-TOF MS analysis of recombinant *Eg*KUs: results for *Eg*KU-3. Mass spectrometry analyses of free thiols and disulphide bonds were carried out as described in Calvete [64]. Three samples were examined for each purified recombinant *Eg*KU: i) untreated (A); ii) denatured with guanidinium hydrochloride and treated with iodoacetamide (IA), to assess the presence of free thiols (B); iii) denatured with guanidinium hydrochloride, reduced with DTT and treated with IA to confirm the presence of 6 Cys residues (C). Predicted MH^+^ values for *Eg*KU-3 are as follows: untreated *Eg*KU-3 (3 Cys-Cys) = 9819 Da; reduced and alkylated *Eg*KU-3 = 10167 Da.
(TIF)

S1 Table cDNA and gene sequence data from *Eg*KU-1-*Eg*KU-8. (PDF)
