# Supplementary material for: Functional diversity of secreted cestode Kunitz proteins: Inhibition of serine peptidases and blockade of cation channels
Source: PLoS Pathog. 2017 Feb 13;13(2):e1006169. doi: 10.1371/journal.ppat.1006169 (PMC5325619; doi:10.1371/journal.ppat.1006169)
Supplement: S1 Dataset — (A) MALDI-TOF mass spectrum of peptides generated by tryptic digestion of cyst fluid proteins recovered after an affinity purification step using chymotrypsin A. The signals that can be assigned to peptides derived from EgKU-8 or EgKU-3 are indicated (refer to Fig 10C). The signal of m/z 1074.54 corresponds to EgKU-8 sequence LPLDPGFcR (theoretical MH+ 1074.53); the signal of m/z 1164.52 to EgKU-3 sequence EQcELLcGR (theoretical MH+ 1164.51); and the signal of m/z 1491.63 to EgKU-8 sequence WGFHQESGEcVR (theoretical MH+ 1491.64); c indicates carbamidomethyl Cys. (B) MS/MS analysis of tryptic peptides from EgKU-3 and EgKU-8. The list of theoretical m/z values of fragment ions is shown for each sequence, and the ions detected in the MS/MS spectra are highlighted in bold. a and b ions correspond to N-terminal fragments, and y ions to C-terminal fragments, according to the accepted nomenclature [80,81]. (PDF) [file ppat.1006169.s001.pdf]

A

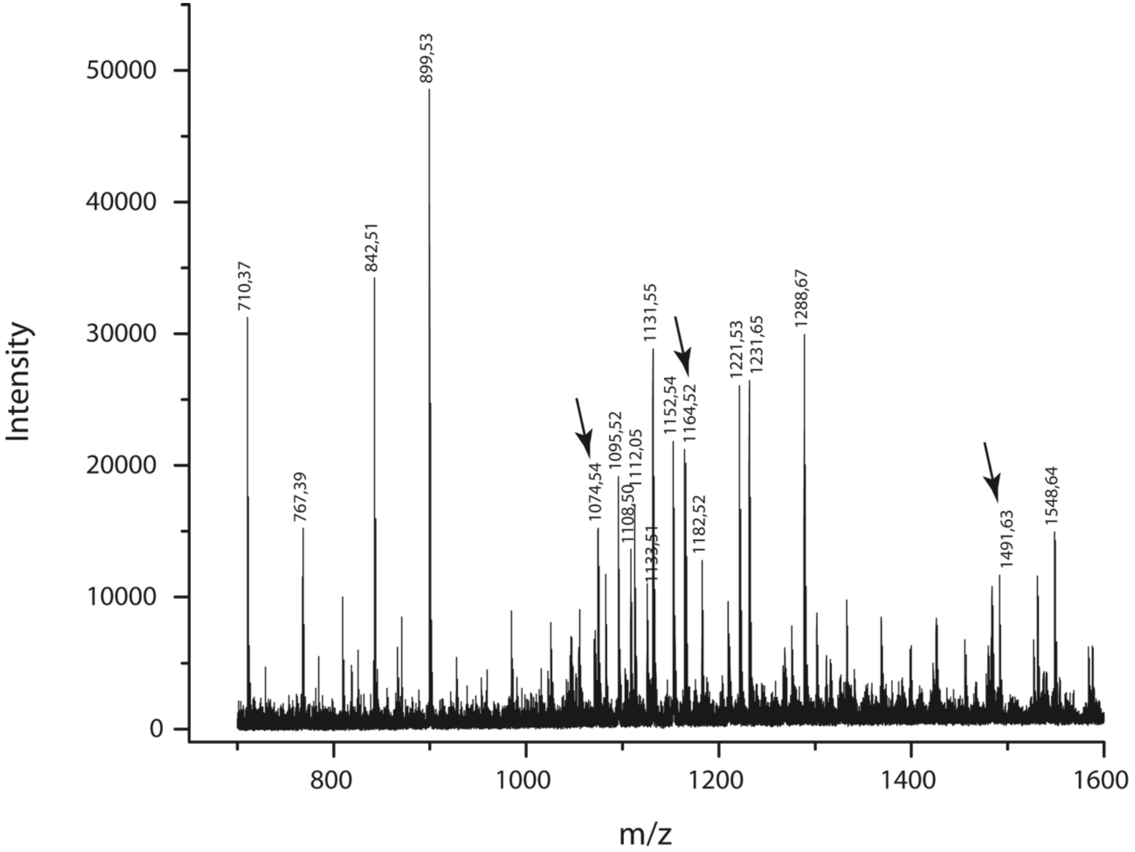

Intensity
50000
40000
30000
20000
10000
0
899,53
842,51
710,37
767,39
1131,55
1288,67
1221,53
1231,65
1152,54
1164,52
1095,52
1112,05
1074,54
1108,50
1133,51
1182,52
1548,64
1491,63
800
1000
1200
1400
1600
m/z

B.

m/z 1074.53 (*Eg*KU-8 peptide **LPLDPGFcR**)

#	a	b	Seq.	y	#
1	86.0964	114.0913	L		9
2	183.1492	211.1441	**P**	**961.4560**	8
3	296.2332	324.2282	L	864.4032	7
4	411.2602	**439.2551**	**D**	751.3192	6
5	508.3129	536.3079	**P**	**636.2922**	5
6	565.3344	593.3293	G	539.2395	4
7	712.4028	740.3977	**F**	**482.2180**	3
8	872.4335	900.4284	**C**	**335.1496**	2
9			**R**	**175.1190**	1

m/z 1164.51 (*Eg*KU-3 peptide **EQcELLcGR**)

#	a	b	Seq.	y	#
1	**102.0550**	130.0499	**E**		9
2	230.1135	**258.1084**	**Q**	**1035.4710**	8
3	390.1442	**418.1391**	**C**	907.4124	7
4	519.1868	**547.1817**	**E**	747.3818	6
5	**632.2708**	**660.2657**	**L**	**618.3392**	5
6	745.3549	773.3498	**L**	**505.2551**	4
7	905.3855	933.3805	**C**	**392.1711**	3
8	962.4070	990.4019	**G**	**232.1404**	2
9			**R**	**175.1190**	1

m/z 1491.64 (*Eg*KU-8 peptide **WGFHQESGEcVR**)

#	a	b	Seq.	y	#
1	**159.0917**	187.0866	**W**		12
2	216.1131	**244.1080**	**G**	1305.5640	11
3	363.1815	391.1765	F	1248.5426	10
4	**500.2405**	**528.2354**	**H**	**1101.4742**	9
5	628.2990	**656.2939**	**Q**	**964.4153**	8
6	757.3416	**785.3365**	**E**	**836.3567**	7
7	844.3737	872.3686	**S**	**707.3141**	6
8	901.3951	**929.3900**	**G**	**620.2821**	5
9	1030.4377	**1058.4326**	**E**	563.2606	4
10	1190.4684	**1218.4633**	**C**	**434.2180**	3
11	1289.5368	1317.5317	V	274.1874	2
12			**R**	**175.1190**	1
