# Supplementary figures and images for: Functional diversity of secreted cestode Kunitz proteins: Inhibition of serine peptidases and blockade of cation channels
Source: PLoS Pathog. 2017 Feb 13;13(2):e1006169. doi: 10.1371/journal.ppat.1006169 (PMC5325619; doi:10.1371/journal.ppat.1006169)

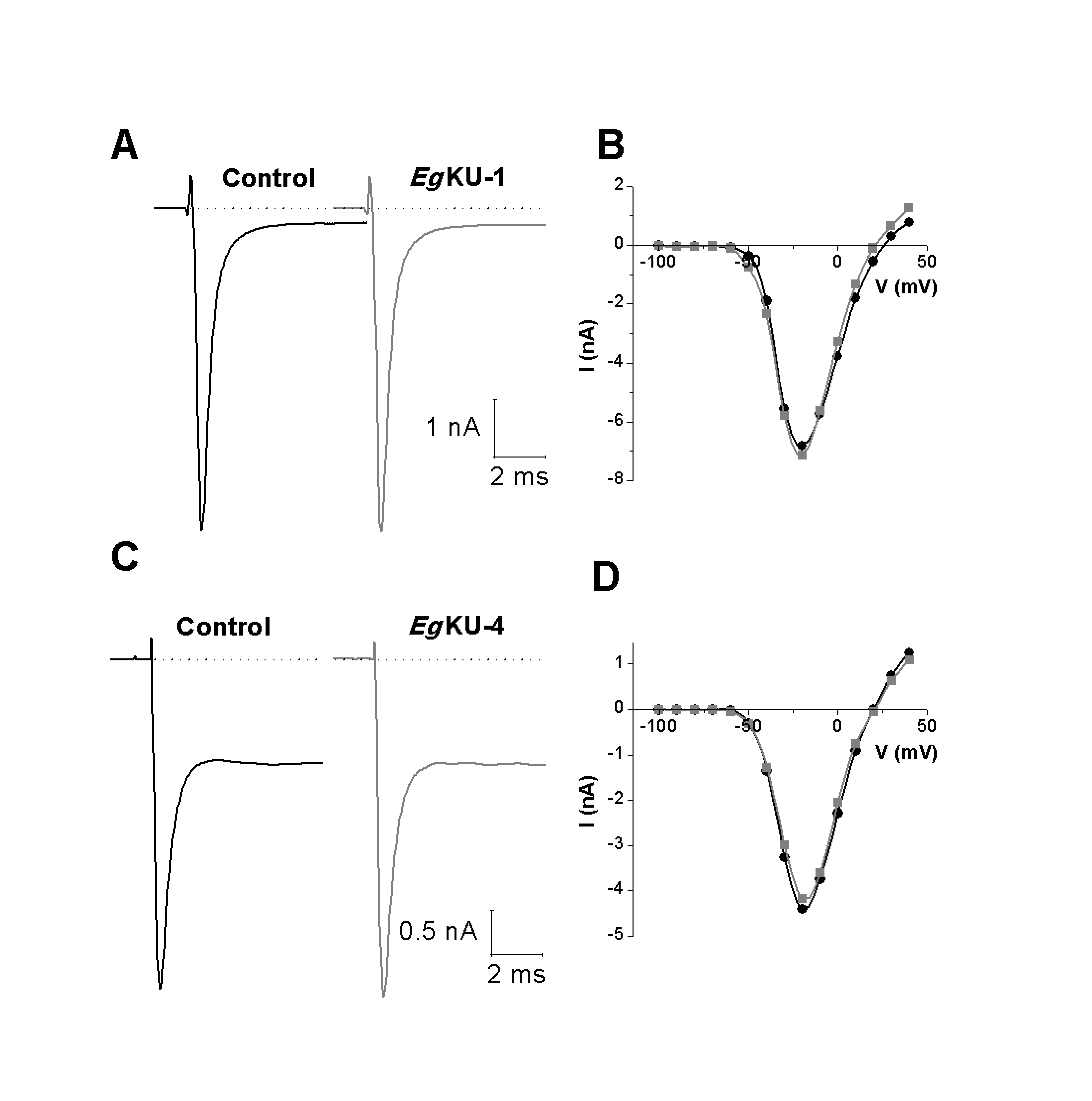

Supplement: S1 Fig — Representative traces showing that the sustained (25 s) perfusion of 100 nM recombinant EgKU-1 (A) or EgKU-4 (C) does not block voltage-activated sodium channels (Nav). (B) and (D) are the current-voltage relationships of the peak Na+ current from the traces in (A) and (C), respectively. The black traces correspond to control conditions and the gray ones after EgKU perfusion. (TIF) [file ppat.1006169.s002.tif]

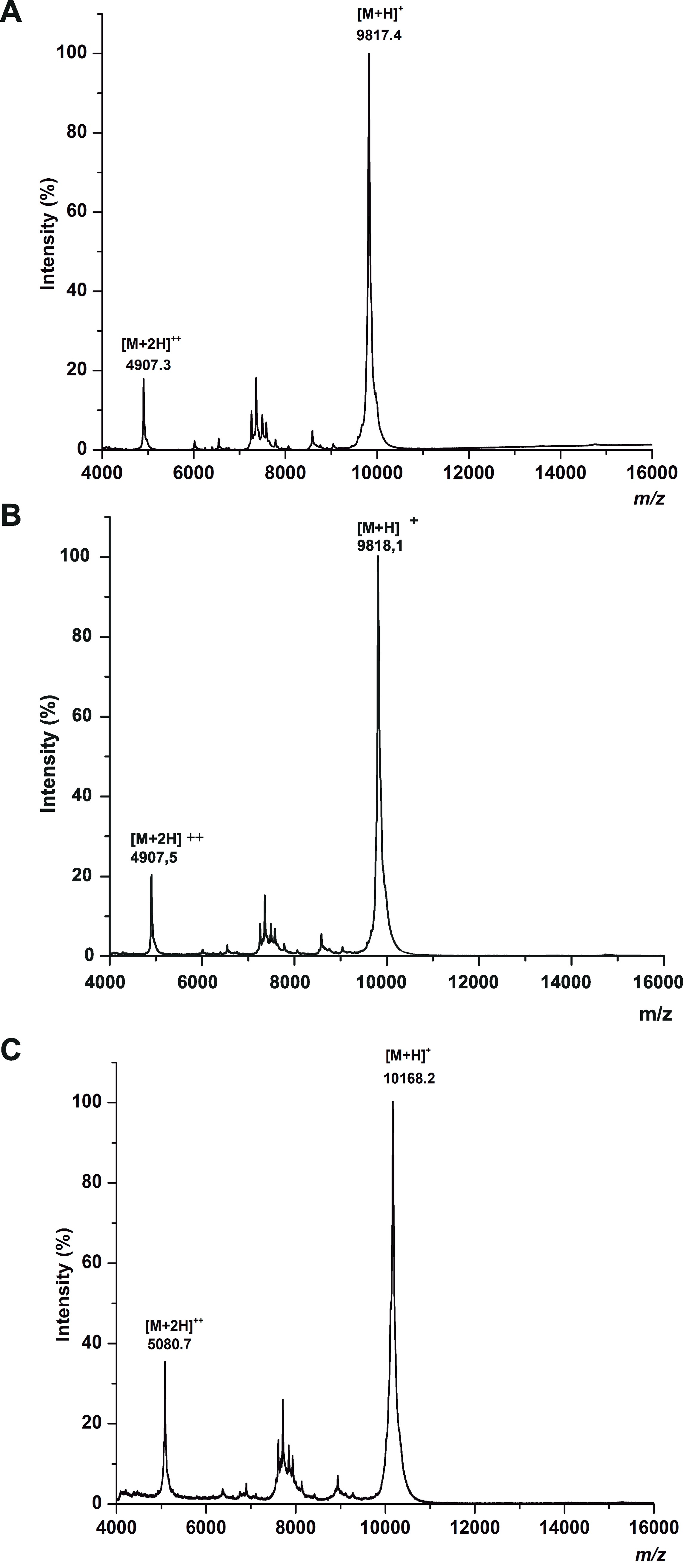

Supplement: S2 Fig — Mass spectrometry analyses of free thiols and disulphide bonds were carried out as described in Calvete [64]. Three samples were examined for each purified recombinant EgKU: i) untreated (A); ii) denatured with guanidinium hydrochloride and treated with iodoacetamide (IA), to assess the presence of free thiols (B); iii) denatured with guanidinium hydrochloride, reduced with DTT and treated with IA to confirm the presence of 6 Cys residues (C). Predicted MH+ values for EgKU-3 are as follows: untreated EgKU-3 (3 Cys-Cys) = 9819 Da; reduced and alkylated EgKU-3 = 10167 Da. (TIF) [file ppat.1006169.s003.tif]
