# Supplementary material for: Functional diversity of secreted cestode Kunitz proteins: Inhibition of serine peptidases and blockade of cation channels
Source: PLoS Pathog. 2017 Feb 13;13(2):e1006169. doi: 10.1371/journal.ppat.1006169 (PMC5325619; doi:10.1371/journal.ppat.1006169)
Supplement: S1 Table — (PDF) [file ppat.1006169.s004.pdf]

S1 Table. cDNA and gene sequence data from *Eg*KU-1-*Eg*KU-8

	cDNA sequence data[a]		Gene sequence data[b]	
	cDNA	EST	WTSC genome	CNHGC genome
***Eg*KU-1**	KY438959	CN650596, CN650753, CN650941, CN652374, CN652433, BI244287	Gene not predicted	Gene not predicted
***Eg*KU-2**	KY438960	CN650229, CN650436, CN650784, CN652059, BI244281	EgrG_000419100	EGR_09268
***Eg*KU-3**	KY438963	BQ173695	EgrG_000534900	EGR_07244
***Eg*KU-4**	KY438961	CN652174	Gene not predicted	Gene not predicted
***Eg*KU-5**	KY438965	CN649551, CN650837	EgrG_000419200	EGR_09269
***Eg*KU-6**	KY438962	CN649590, CN649996	EgrG_000549400	EGR_04958
***Eg*KU-7**	KY438964	CN648947	EgrG_000548800	EGR_10096
***Eg*KU-8**	FJ031017	--	EgrG_000534700	EGR_07243

[a]NCBI accession numbers of cDNA and EST sequences (from GenBank and dbEST, respectively). The assembled ESTs were described in our previous study [3].
[b]Gene ID from the *E. granulosus* genome sequences produced at the Wellcome Trust Sanger Centre (WTSC; [5]) and the Chinese National Human Genome Center (CNHGC; [35]).
